# RECQ1 Promotes Stress Resistance and DNA Replication Progression Through PARP1 Signaling Pathway in Glioblastoma

**DOI:** 10.3389/fcell.2021.714868

**Published:** 2021-07-26

**Authors:** Jing Zhang, Hao Lian, Kui Chen, Ying Pang, Mu Chen, Bingsong Huang, Lei Zhu, Siyi Xu, Min Liu, Chunlong Zhong

**Affiliations:** ^1^Department of Neurosurgery, Shanghai East Hospital, School of Medicine, Tongji University, Shanghai, China; ^2^Institute for Advanced Study, Tongji University, Shanghai, China

**Keywords:** genomic stability, drug resistance, DNA replication stress, fork reversal, RECQ1, PARP1

## Abstract

Glioblastoma (GBM) is the most common aggressive primary malignant brain tumor, and patients with GBM have a median survival of 20 months. Clinical therapy resistance is a challenging barrier to overcome. Tumor genome stability maintenance during DNA replication, especially the ability to respond to replication stress, is highly correlated with drug resistance. Recently, we identified a protective role for RECQ1 under replication stress conditions. RECQ1 acts at replication forks, binds PCNA, inhibits single-strand DNA formation and nascent strand degradation in GBM cells. It is associated with the function of the PARP1 protein, promoting PARP1 recruitment to replication sites. RECQ1 is essential for DNA replication fork protection and tumor cell proliferation under replication stress conditions, and as a target of RECQ1, PARP1 effectively protects and restarts stalled replication forks, providing new insights into genomic stability maintenance and replication stress resistance. These findings indicate that tumor genome stability targeting RECQ1-PARP1 signaling may be a promising therapeutic intervention to overcome therapy resistance in GBM.

## Introduction

Glioblastoma (GBM) is the most common, aggressive adult primary brain tumor and is associated with profound genomic heterogeneity and limited cure development to date (Stupp et al., 2017). The capacity for DNA replication damage repair and genome stability maintenance during tumor proliferation is thought to contribute to therapy resistance. The DNA replication machinery must duplicate genomic information, overcome numerous obstacles established by endogenous and exogenous replication stress that impair replication fork progression and DNA synthesis during DNA replication (Zeman and Cimprich, 2014). Under replication stress, protection of DNA replication is critical for the maintenance of genomic integrity, driving functional “protection” for the survival of rapidly dividing tumor cells, which also leads to resistance to cancer drug therapy. In addition, cells suffer uncontrolled degradation and replication fork collapse, promoting DNA damage and genomic instability.

Human RecQ helicases (WRN, BLM, RECQ4, RECQ5, and RECQ1) play critical roles in protecting and stabilizing the genome (Hickson, 2003; Opresko et al., 2004). Depending on their ability to unwind various DNA structures, multiple cellular functions have been associated with RecQ proteins, including those with roles in stabilizing damaged DNA replication forks, telomere maintenance, and homologous recombination (Sharma et al., 2006; Bohr, 2008; Ouyang et al., 2008). Of the five human RecQ families, three are genetically linked to cancer syndromes and premature diseases, such as Bloom’s syndrome (BLM gene mutations), Werner’s syndrome (WRN gene mutations), Rothmund-Thomson syndrome (RTS), RAPADILINO, and Baller-Gerold syndrome (caused by mutation of RECQ4) (Ellis et al., 1995; Yu et al., 1996; Kitao et al., 1999; Siitonen et al., 2003). As the most abundant of the five human RecQ proteins, although the clinical importance of RECQ1 has only been partially revealed, the currently mysterious, unknown, unique, and important roles of RECQ1 in cellular DNA metabolism need to be discovered.

RECQ1 is overexpressed in rapidly dividing cells and multiple cancer cells, including human GBM, ovarian cancer, and hypopharyngeal cancer (Mendoza-Maldonado et al., 2011; Viziteu et al., 2017; Debnath and Sharma, 2020). However, the function and mechanism of RECQ1 activity in cancer proliferation remain unknown. We hypothesized that RECQ1 plays a particularly important role in facilitating DNA replication stress in cancer cells that undergo rapid proliferation. RECQ1 has recently been reported to confer specific oncogene effects, including cell cycle progression in the S-phase, to tumors, and systemic depletion of RECQ1 has been shown to prevent tumor growth in murine models (Futami et al., 2008; Arai et al., 2011; Mendoza-Maldonado et al., 2011), but a direct role for RECQ1 in DNA replication and cellular processes has not been identified.

In this study, we demonstrate that RECQ1 plays an essential role in DNA replication fork protection and thus maintains genome stability in response to replication stress. RECQ1 is located at replication sites and associates with proliferating cell nuclear antigen (PCNA) and physically interacts with poly[ADP-ribose] polymerase 1 (PARP1), which binds to unresected stalled DNA replication forks and recruit XRCC1 to mediate repair and promote replication restart, thereby protecting replication fork stability (Ying et al., 2016; Thakar et al., 2020). We show that RECQ1 depletion results in increased nascent strand degradation and fork stalling, DNA double-strand breaks (DSBs) and a decreased cell proliferation rate under replication stress, supporting the notion that RECQ1 plays a specific role in the maintenance of genomic stability. In addition, RECQ1-depleted cells are hypersensitive to methyl methanesulfonate (MMS) and temozolomide (TMZ) treatment. Glioblastoma is the most common and aggressive malignant histotype of brain tumor, and patients with glioblastoma have a poor prognosis (Lefranc et al., 2005; Stupp et al., 2009). Although considerable advancements in glioblastoma treatment have been investigated in recent years, new therapeutic strategies are still urgently needed. Considering these data, we propose that RECQ1 regulates PARP1 protein function and protects DNA replication fork stability to maintain genomic stability under replication stress conditions and contributes to the drug resistance of GBM cells. This study provides a novel approach to GBM suppression whereby mediating the DNA repair RECQ1-PARP1 signaling pathway function inhibits DNA replication.

## Materials and Methods

### Cell Culture

M059K, U251, U87MG, and RPE1 cells were purchased from American Type Culture Collection (ATCC). Cells free of mycoplasma contamination were maintained in Dulbecco’s modified Eagle’s medium (DMEM) or a 1:1 mixture of DMEM and Ham’s F12 medium supplemented with 2.5 mM L-glutamine, 15 mM HEPES, 0.5 mM sodium pyruvate, 1.2 g/L sodium bicarbonate, 0.05 mM non-essential amino acids and 10% fetal bovine serum. Cells were cultured at 37°C in a humidified atmosphere containing 5% CO2.

### siRNA and Transfection

Cells were seeded in six-well plates and transfected with indicated siRNA. The siRNA-targeting sequences used were: siRECQ1: (Dharmacon SMARTpool, GAGCUUAUGUUACCAGUUA, CUACGGCUUUGGAGAUAUA, GAUUAUAAGGCACUUGG UA, GGGCAAGCAAUGAAU AUGA). siPARP1: UUCUCCGA ACGUGUCACGUTT, GAGGAAGGUAUCAACAAAUTT, GA GCACUUCAUGAAAUUAUTT, GAGACCCAAUAGGUUAAU TT. Negative Control siRNA and siPARP1 (GAGGAAGGUA UCAACAAA UTT) (Qiagen). PARP1 expression ORF was purchased from OriGene Technologies, transfection by using Lipofectamine transfection reagent (L3000015, Thermo Fisher Scientific) according to the manufacturer’s instructions. Cells were harvested on day 4 after transfection for further analyses.

### Antibodies and Reagents

The antibodies anti-RECQ1 (ab151501, 1:200 dilution), anti-53BP1 (ab175933, 1:200 dilution), anti-RPA (ab2175, 1: 1000 dilution), anti-PARP1 (ab191217, 1:1000 dilution) and anti-BrdU (ab6326, 1:200), anti-RPA2-pS4/S8 (ab87277, 1:200 dilution) were purchased from Abcam. Antibodies anti-γH2AX (2577, 1:800 dilution), α-Tubulin (2144, 1:1000 dilution) and PCNA antibody (2586, 1:200 dilution) were purchased from Cell Signaling. Mouse anti-BrdU (347580, 1:40) was purchased from BD Biosciences. AF647 (A-21247, 1:1000) and AF488 (A-11001, 1:1000) were purchased from Thermo Fisher Scientific. BMN673 (S7048, Talazoparib) was purchased from Selleck Chemicals.

### Immunofluorescence Staining

M059K cells were cultured in 35 mm plates and transfected with siRECQ1, siPARP1 or PARP1 OE, followed indicated treatment. Then cells were washed with PBS and fixed with 4% formaldehyde. Cells were permeabilized with Triton X-100 (0.05%) for 10 min, blocked with 3% BSA in PBS and then incubated overnight at 4°C with primary antibodies. Next, cells were washed and incubated with AF488 or AF647-conjugated secondary antibody. Finally, cells were washed with PBST for three times and stained with DAPI for 10 min at RT. Images of the mounted slides were acquired with a Zeiss Axiovert 200 M microscope.

### DNA Fiber Spreading Analysis

DNA fiber spreading assays were performed as followed. Briefly, cells were transfected with siRECQ1, siPARP1 or PARP1 OE, followed by incubation with 10 μM CldU for 30 min and then with 100 μM IdU for another 30 min. For treatment, cells were exposed to 2 mM HU or 50 μM MMS before or after IdU incubation. Cells were then suspended in PBS, and ∼200 cells placed on a glass microscope slide (Newcomer Glass) and 10 μl of lysis buffer (0.5% SDS in 200 mM Tris–HCl pH 7.5, 50 mM EDTA) added. DNA fibers were spread and fixed in methanol: acetic acid (3:1), denatured with 2.5 M HCl for 1 h, neutralized in 0.4 M Tris–HCl pH 7.5 for 5 min, washed in PBS, and immunostained using anti-BrdU primary and corresponding secondary antibodies. The slides were mounted in ProLong Gold Anti-fade Mounting medium. Images were acquired using a Zeiss Axiovert 200 M microscope at ×63 magnification with the Axio Vision software packages (Zeiss).

### *In situ* Proximity Ligation Assay (PLA)

Cells were grown on 35 mm MatTek glass bottomed plates followed by transfection with siRECQ1 in the presence or absence of MMS, then cells were incubated with 0.1% formaldehyde for 5 min and treated twice total 10 min with CSK-R buffer (10 mM PIPES, pH 7.0, 100 mM NaCl, 300 mM sucrose, 3 mM MgCl2, 0.5% Triton X-100, 300 μg/ml RNAse), and fixed in 4% formaldehyde in PBS (W/V) for 10 min at RT, followed by incubation in pre-cold methanol for 20 min at −20°C. After washing with PBS for three times, cells were treated with 100 μg/ml RNAse in 5 mM EDTA buffer for 30 min at 37°C. *In situ* PLA was performed using the Duolink PLA kit (Sigma-Aldrich) according to the manufacturer’s instructions. Briefly, the cells were blocked for 30 min at 37°C and incubated with primary antibodies for 30 min at 37°C. Following three times washing with PBST (phosphate buffered saline, 0.1% Tween), anti-mouse PLUS and anti-rabbit MINUS PLA probes were coupled to the primary antibodies for 1 h at 37°C. After three times washing with buffer A (0.01 M Tris, 0.15 M NaCl, and 0.05% Tween-20) for 5 min, PLA probes were ligated for 30 min at 37°C. After three times washing with buffer A, amplification using Duolink *In Situ* Detection Reagents (Sigma) was performed at 37°C for 100 min. After amplification, the plates were washed for 5 min three times with wash buffer B (0.2 M Tris 0.1 M NaCl). Finally, they were coated with mounting medium containing DAPI (Prolong Gold, Invitrogen) and imaged using a Zeiss Axiovert 200 M microscope.

### Cell Survival Assay and Cell Viability Assay

Cell survival fraction was assessed by evaluating the colony-forming ability. In brief, M059K and U251 cells were seeded in six-well plates (500 cells per well) after transfection with siRECQ1, siNC, in the presence of different concentration of TMZ and were subsequently incubated for 10 days to allow colonies to develop. Cells were finally fixed with cold methanol, and the colonies were stained with crystal violet (in a 100% methanol solution) for manual counting.

The viability of M059K and U251 cells was assessed with a Cell Counting Kit-8 (CCK8) kit (DOJINDO Laboratories) according to the manufacturer’s instructions. Cells were seeded in 96-well plates and cultured in a 37°C incubator for up to 4 days after treatment, and the OD at 450 nm was measured. All cell-based assays were performed in at least triplicate.

### Nuclear Morphology Assay

The chromosome breakage assay was performed as described previously (van der Crabben et al., 2016). In brief, M059K cells were treated with siRECQ1. After 4 days of culture, cells were incubated with hypotonic solution (0.56% KCl) at room temperature for 30 min and then in a 37°C water bath for 5 min. Fixation with pre-cooled fixation buffer (methanol: acetic acid = 3:1) was repeated three times, and a dropper was used to place cells onto a clean slide. Spread cells were incubated at 55°C overnight and stained with Giemsa solution (GS-500, Sigma) for image acquisition of aberrant chromosomes with a Zeiss Axiovert 200 M microscope.

### Western Blot Analysis

Cells transfected with siRECQ1, siPARP1, or PARP1 OE were harvested and lysed in lysis buffer (50 mM Tris–HCl (pH 7.4), 0.15 M NaCl, and 1% Triton X-100 in PBS, supplemented with protease and phosphatase inhibitors) on ice for 30 min. Proteins were separated by SDS-PAGE on an 8–16% gel (Invitrogen) and transferred to a PVDF membrane. The membranes were blocked in 5% dry milk in 0.1% Tween-20 in PBS and detected with indicated antibodies. After incubation with horseradish peroxidase (HRP)-conjugated secondary antibodies (Bio-Rad), then immunoreactions were visualized using ECL western blot detection reagents (Pierce Biotechnology) and Image Lab 5.1 gel densitometry analysis system. ImageJ software (version 1.8.0.) was used to analyze protein bands.

### Statistical Analysis

Statistical significance of PLA experiments was analyzed using the Mann–Whitney rank-sum test and expressed as mean ± SEM values. Fiber patterns and immunoblotting were analyzed using a two-sided unpaired *t*-test, and the exact *P*-values are given in each case. These data are expressed as the mean and standard deviation (mean ± SD) values. Statistical analysis was performed using Student’s independent *t*-test, and two-sided *p*-values. All experiments data were calculated via GraphPad Prism 8.4.2 software to assess the significance of differences between experimental groups. For all tests: significant: *P* < 0.05, NS (not significant): *P* > 0.05. All experiments were performed at least three times.

## Results

### RECQ1 Depletion Causes Nascent Strand Degradation Under Replication Stress

**FIGURE 1:**
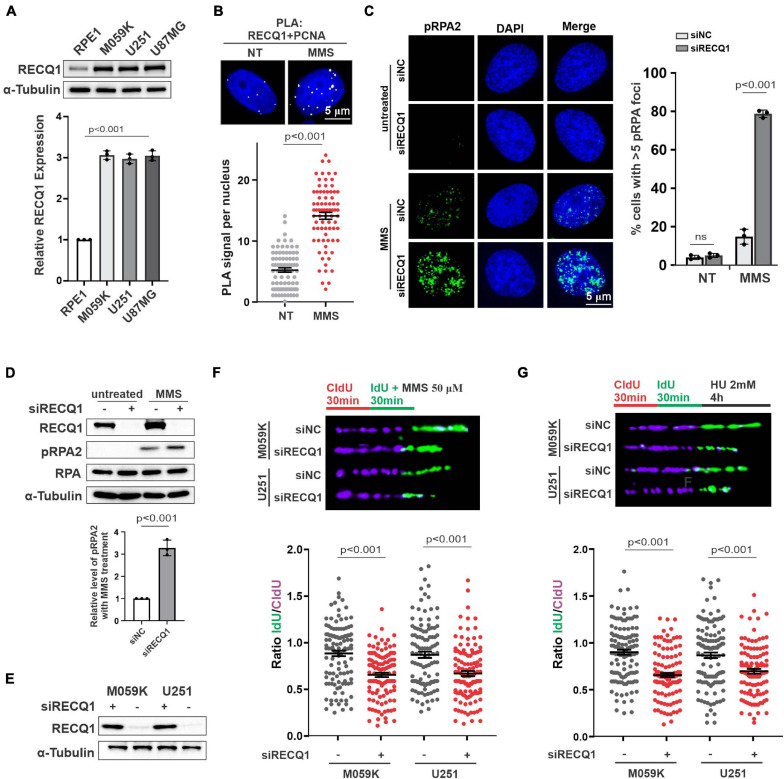
RECQ1 is critical for replication fork restoration in response to replication stress. **(A)** Whole-cell lysates from different cell lines were analyzed by western blotting using indicated antibodies against RECQ1 and α-Tubulin, indicating RECQ1 is overexpressed in GBM cell lines. **(B)** Detection of RECQ1-PCNA interaction was carried out by PLA labeling in M059K cells treated with or without 50 μM MMS. Representative images are shown. Scale bars, 5 μm. The scatterplot displays quantification of the PLA signals per nucleus from three independent experiments. Data are mean ± SEM. **(C)** M059K cells were transfected with siNC or siRECQ1 for 48 h and then treated with the MMS or not. Immunofluorescence labeling was performed to detect level of pRPA2 for ssDNA accumulation analysis. Quantitation of pRPA2 was presented from three independent replicates. Data are mean ± SD. **(D,E)** Whole-cell lysates from different cell lines with MMS treatment or not were analyzed by western blotting using the indicated antibodies. **(F,G)** Schematic of the CldU/IdU pulse-labeling analysis used to investigate nascent Strand degradation upon MMS or HU treatment in M059K and U251 cells transfected with siRECQ1 targeting RECQ1 for 48 h. Representative images of CldU and IdU replication tracks (top) and scatterplot of IdU/CldU-tract length ratios (bottom) for replication forks are shown. Fiber evaluated from at least 100 events from three independent experiments. Data are mean ± SEM. A two-sided Mann–Whitney rank-sum test was used to determine if differences were significant **(B,F,G)**. A two-sided unpaired *t*-test was used to calculate *P*-values **(C)**. Significant: *p* < 0.05; NS, not significant: *P* > 0.05.

Given the characteristic RECQ1 overexpression in glioblastoma patient tumor tissue, RECQ1 has been suggested to play a probable role in GBM tumorigenesis and progression. To determine whether the glioblastoma cell lines used in this study express high levels of RECQ1, the RECQ1 protein levels in the M059K, and U251MG glioblastoma cell lines were compared to those in the RPE1 non-cancerous retina pigmented epithelium cell line by western blot assay (Figure 1A). A quantitative analysis confirmed that M059K, U251 and U87MG glioblastoma cells express a higher level of RECQ1 than RPE1 cells, which is in line with previous studies.

To investigate the function of RECQ1 in GBM cell proliferation, we first carried out proximity ligation assays (*in situ* PLA) using antibodies against RECQ1 and PCNA, which bind and indicate DNA replication intermediates during DNA replication. We detected PLA signals in untreated M059K cells but significantly increased PLA signals in cells treated with methyl methanesulfonate (MMS), an alkylating agent that transiently arrests fork progression by causing replication fork stalling. The increased nuclear PLA signals suggested an enhanced interaction of RECQ1 and replication sites in response to replication stress (Figure 1B). RPA-coated ssDNA is a universal feature of stalled replication forks that presents a recruiting platform for downstream repair factors and checkpoint kinases, such as ATR and CHK1 (Marechal and Zou, 2013). Therefore, phosphorylation of RPA2 at serine 4 and serine 8 (S4/S8) is a commonly used marker of DNA replication stress (Lossaint et al., 2013; Fugger et al., 2015). We compared the impact of RECQ1 deficiency on RPA2 phosphorylation levels upon MMS treatment. As shown in Figure 1C, RECQ1 depletion resulted in a dramatic increase in RPA2 phosphorylation in cells exposed to MMS, indicating that RECQ1 inhibited ssDNA accumulation and RPA binding to chromatin in response to replication stress. The increased expression of RPA2 phosphorylation was confirmed by western blot analysis with RECQ1-depleted M059K cells, showing that RECQ1 stabilizes stalled replication forks by limiting ssDNA formation (Figure 1D).

Prolonged fork stalling upon MMS treatment has been previously shown to result in fork collapse by uncontrolled nucleolytic degradation and ultimately results in replication-coupled DSB formation (Toledo et al., 2013). In the present study, western blot analysis showed decreased expression of RECQ1 in cells with RECQ1 siRNA transfection (Figure 1E). To gain further insights into the mechanism of RECQ1 depletion-induced ssDNA accumulation during DNA synthesis under replication stress conditions, we performed a DNA spreading assay. Nascent replication strands were sequentially labeled with chlorodeoxyuridine (CldU) and iododeoxyuridine (IdU) for 30 min, and then, they were subjected to 50 μM MMS treatment. RECQ1 deficiency resulted in a dramatic shortening of nascent replication strands in two distinct GBM cell lines, M059K, U251, and U87MG cells (Figure 1F), indicating that the forks damaged upon MMS exposure underwent excessive nuclease degradation upon RECQ1 depletion. A similar phenotype was observed in GBM cells treated with hydroxyurea (HU), a drug that causes fork stalling by depleting the pool of nucleotides available for DNA synthesis (Figure 1G). In light of these findings, we proposed that the helicase RECQ1 can prevent ssDNA formation and nascent strand degradation upon replication stress in GBM cells.

### RECQ1 Depletion Inhibit GBM Cells Growth Upon Replication Stress

**FIGURE 2:**
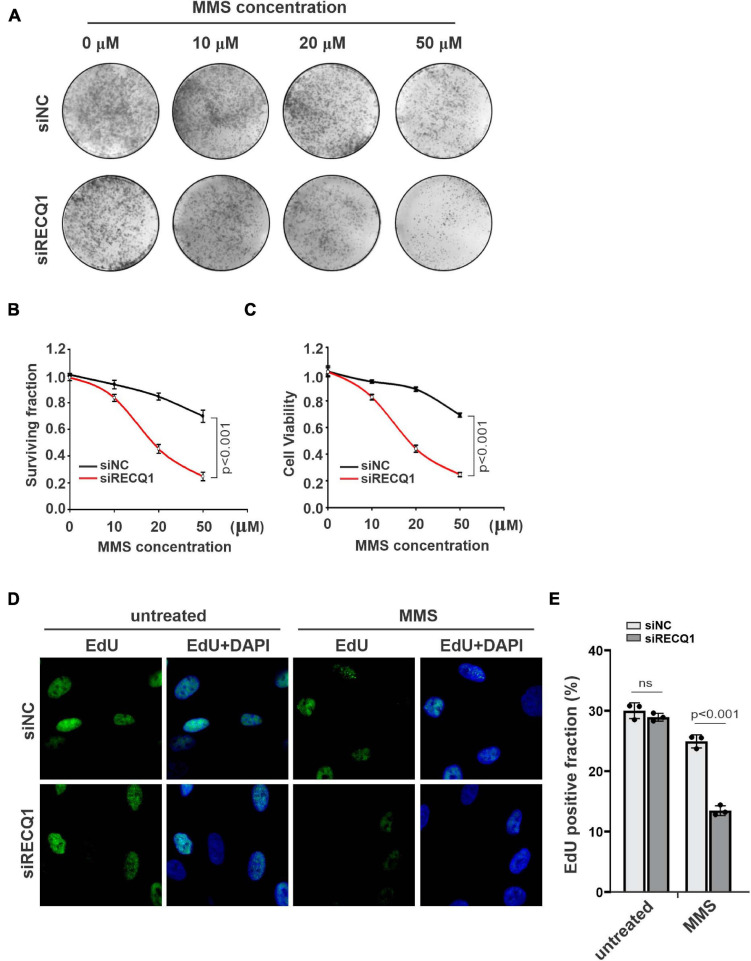
RECQ1 depletion impaired GBM cells cellular progression. **(A)** Colony formation assay of M059K cells treated with different concentration of MMS after transfection with siNC or siRECQ1. Representative images are shown. **(B)** Quantification are represented as means ± SD from at least three independent experiments. **(C)** The viability of siRECQ1 transfected M059K cells was measured with a CCK8 kit. Data are represented as means ± SD from at least three independent experiments. **(D)** Cell proliferation was measured by an EdU labeling of immunofluorescence. **(E)** Quantification are represented as means ± SD from at least three independent experiments. A two-sided unpaired *t*-test was used to calculate *P*-values. Significant: *p* < 0.05; NS, not significant: *P* > 0.05.

To investigate the role of RECQ1 in promoting glioblastoma cell growth and proliferation, we assayed the colony formation properties of M059K cells treated with different concentrations of MMS. Compared with cells transfected with control siRNA (causing an approximate 20% reduction in colony formation), the cells transfected with RECQ1 siRNA exhibited an approximate 80% reduction in colony formation (Figures 2A,B). Similar results were also found with a viability CCK-8 cell viability assay (Figure 2C). The cell survival curves with increasing MMS concentrations indicated that RECQ1-deficient cells were hypersensitive to MMS treatment and further confirmed the impairment of the repair function of RECQ1 in DNA replication fork progression with accumulated DNA damage caused by increased replication stress. To provide further evidence for suppressed cell proliferation due to RECQ1 deficiency, we performed EdU staining of RECQ1-depleted M059K cells and showed that the number of EdU positive cells reduced by 50% (Figures 2D,E) indicating a slowed cellular progression upon RECQ1 depletion under replication stress conditions. These data confirmed that the downregulation of RECQ1 significantly suppressed the proliferation capacity of GBM cells by interfering with DNA synthesis and DNA replication fork progression under DNA replication stress. Thus, underlining the potential implications of these findings for GBM tumor growth, depletion of RECQ1 resulted in a progressive proliferative defect in GBM cells.

### RECQ1 Deficiency in Glioblastoma Cells Results in Accumulated DNA Damage

**FIGURE 3:**
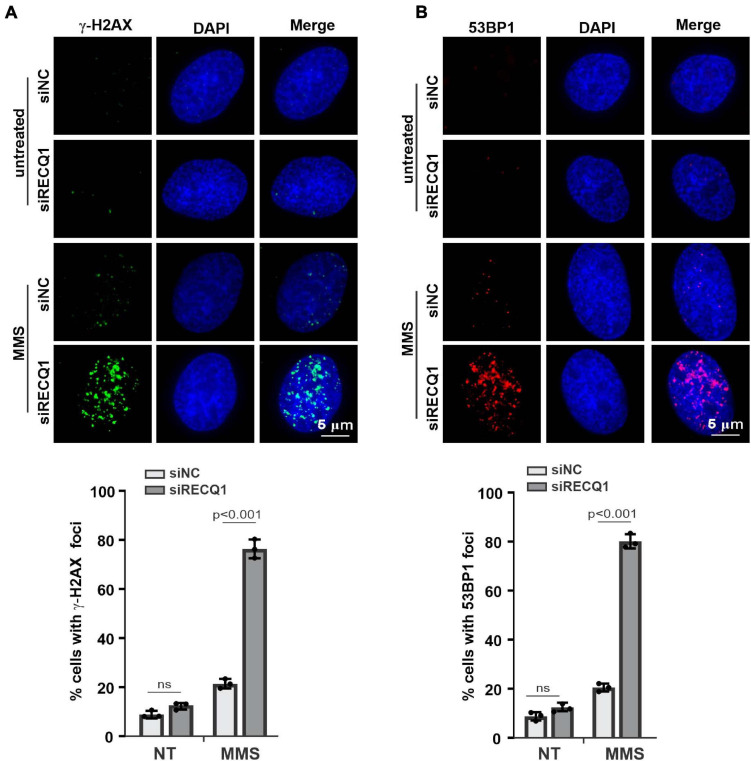
RECQ1 deficiency leads to GBM cells DSB repair defects. **(A,B)** Treatment of M059K cells with siRECQ1 exposed to MMS resulted in DSBs accumulation, as indicated by nuclear γ-H2AX and 53BP1 staining. Quantification are represented as means ± SD from at least three independent experiments. A two-sided unpaired *t*-test was used to calculate *P*-values. Significant: *p* < 0.05; NS, not significant: *P* > 0.05.

RecQ helicases have been previously reported to associate with the stabilization and restarting of damaged DNA replication forks in response to multiple replication stresses (Brosh and Bohr, 2007). Long-term fork stalling and failure to restart damaged forks can lead to DNA breaks (Andreassen et al., 2006). Given the previous studies that RECQ1-deficient cells showed a mildly increased level of DNA damage with homologous recombination (HR) repair impairment under the untreated condition (Mendoza-Maldonado et al., 2011), we sought to evaluate whether RECQ1 depletion can trigger DNA damage upon MMS treatment. We analyzed the extent of spontaneous γ-H2AX and 53BP1 foci formation in M059K cells transfected with RECQ1 siRNA or siNC in the presence or absence of MMS; H2AX and 53BP1 are well-known markers of DNA double-strand breaks in damaged cells. Immunofluorescence analysis indicated that RECQ1 depletion resulted in a dramatic increase in γ-H2AX and 53BP1 foci formation in M059K cells exposed to MMS compared to unchallenged cells, confirming that the depletion of RECQ1 was associated with defects in DSB repair (Figures 3A,B). Approximately 80% of the RECQ1-deficient cells harbored more than five γ-H2AX and 53BP1 foci per nucleus, compared to approximately 20% in the RECQ1-proficient cells, implying that the DNA repair capacity of RECQ1-deficiency cells was diminished upon MMS treatment. There was a much lower detectable percentage of cells with DSB damage among the untreated cells, indicating that specific DNA damage repair is mediated under different conditions. Increased DNA damage is often associated with growth inhibition, which is specific to cancer cells, as they rapidly proliferate and exhibit more DNA damage (Gangopadhyay et al., 2014; Qu et al., 2014). Therefore, the results of DNA damage accumulation demonstrated the suppressed growth of GBM cells with RECQ1 deficiency in response to MMS-induced replication stress.

### RECQ1 Depletion Inhibit the Growth of GBM Cells by Impairing PARP1 Function

**FIGURE 4:**
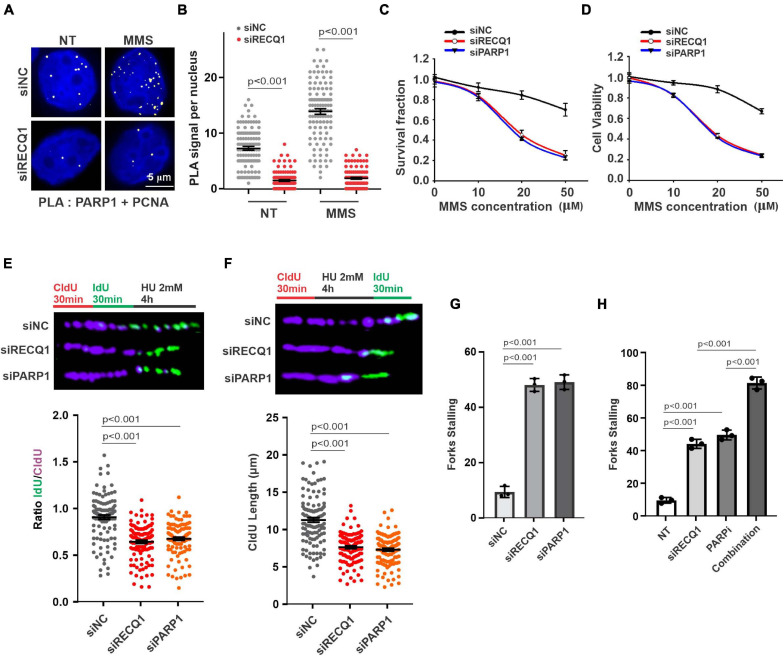
RECQ1 function on GBM cells growth by mediating PARP1 pathway. **(A)** Detection of PARP1-PCNA interaction was carried out by PLA labeling in M059K cells treated with or without 50 μM MMS after transfection with siNC or siRECQ1. Representative images are shown. Scale bars, 5 μm. **(B)** The scatterplot displays quantification of the PLA signals per nucleus from three independent experiments. Data are mean ± SEM. **(C)** Quantification of colony formation assay of cells transfected with siNC, siRECQ1 or siPARP1 exposed to different concentration of MMS. **(D)** The viability of siRECQ1 transfected M059K cells was measured with a CCK8 kit. **(E)** Schematic of the CldU/IdU pulse-labeling analysis used to investigate replication fork degradation upon HU treatment in M059K cells transfected with siRECQ1 or siPARP1 for 48 h. Representative images of CldU and IdU replication tracks (top) and scatterplot of IdU/CldU-tract length ratios (bottom) for replication forks are shown. Fiber evaluated from at least 100 events from three independent experiments. Data are mean ± SEM. **(F–H)** Schematic of an alternative CldU/IdU pulse-labeling protocol to investigate fork degradation and fork stalling upon HU treatment in M059K cells of CldU tracking length and stalled forks are assayed. Fiber evaluated from at least 120 events from three independent experiments. Data are mean ± SEM. A two-sided Mann–Whitney rank-sum test was used to determine if differences were significant **(B,E,F)**. A two-sided unpaired *t*-test was used to calculate *P*-values **(C,D)**. Significant: *p* < 0.05; NS, not significant: *P* > 0.05.

Earlier studies demonstrated that RECQ1 is directly associated with PAPR1 and contributes to fork restoration (Berti et al., 2013). To investigate the mechanism of RECQ1 in protecting replication forks upon DNA replication stress in GBM cells, we evaluated the response of PARP1 to replication stress. In stark contrast to the finding that RECQ1-deficient cells activated PARP1 in a specific response to H_2_O_2_ treatment, PARP1 was shown to be significantly recruited to the replication site, as indicated by PLA assay performed to test the interaction between PAPR1 and PCNA upon specific MMS treatment (Figures 4A,B). These results suggested the possibility that RECQ1 protects damaged forks by regulating PARP1 function and performs different mechanisms in response to various types of DNA damage. Survival and viability analyses validated the finding showing that cells deficient in RECQ1 and PARP1 were sensitive to replication stress induced by MMS (Figures 4C,D).

M059K cells were next transfected with PARP1 siRNA and scramble siNC prior to a DNA replication progression assay. PARP1-deficient cells contained shortened IdU strands, representing unprotected damaged replication forks similar to those captured in cells lacking RECQ1 in the presence of HU (Figure 4E). To evaluate the importance of PARP1 in the protection of stalled DNA replication forks, cells were exposed to long-term HU exposure before IdU labeling was performed a second time. The results from a DNA fiber analysis showed that nascent CldU strands in PARP1-deficient M059K cells were shortened during replication fork stalling, compared with the strands in PARP1-proficient cells, which was consistent with the observations of RECQ1-deficient cells (Figure 4F). Consistent with the expectation that RECQ1 and PARP1 function in stalled fork restarting, increased fork stalling was observed in both RECQ1- and PARP1-depleted cells (Figure 4G). In addition, RECQ1 depletion increased fork stalling to PARP inhibitor, suggesting a combined effect of RECQ1 and PARP on fork stability under replication stress (Figure 4H). Together, these observations raised the possibility that RECQ1 and PARP1 collaboratively protect stalled DNA forks and maintain cellular proliferation under specific replication stress conditions.

### PARP1 Restored Cell Growth With RECQ1 Depletion Under Replication Stress

**FIGURE 5:**
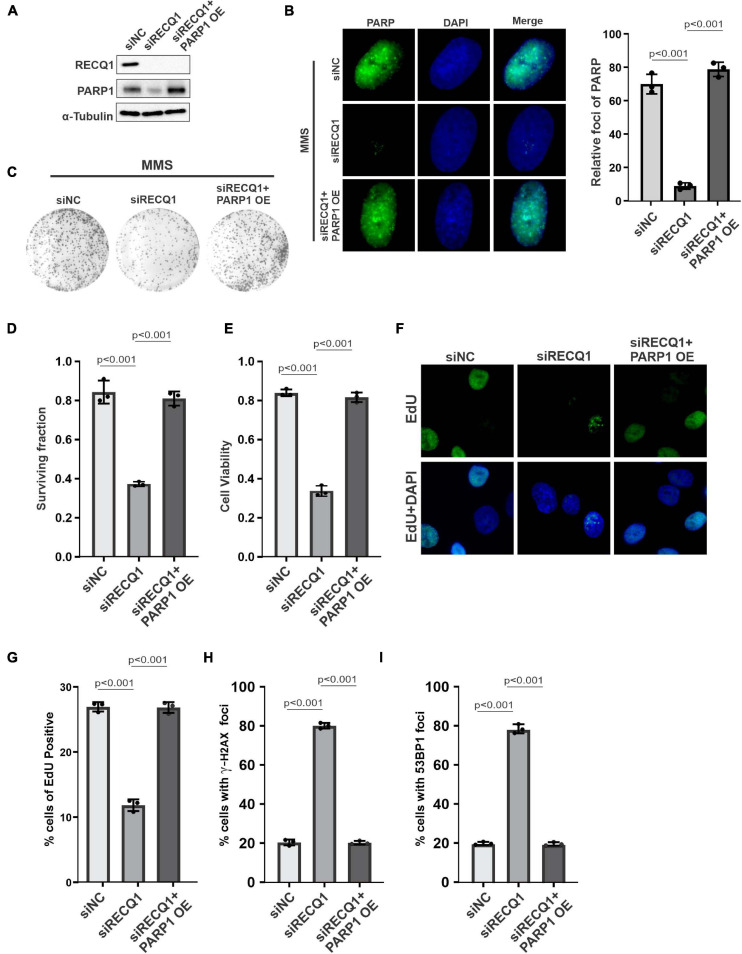
PARP1 compensated RECQ1 dysfunction on GBM cells growth upon replication stress. **(A)** Whole-cell lysates from M059K cells transfected with siNC, siRECQ1, or siRECQ1 and PARP1 OE (overexpression plasmid) were analyzed by western blotting using the indicated antibodies. **(B)** Immunofluorescence indicates PARylation in cells transfected with siNC, siRECQ1, or siRECQ1 and PARP1 OE. **(C)** Representative images of colonies. **(D)** Quantification of colony formation assay of cells transfected with siNC, siRECQ1 or siRECQ1 and PARP1 OE exposed to MMS. **(E)** The viability of transfected M059K cells was measured with a CCK8 kit. **(F,G)** Cell proliferation was measured by an EdU labeling of immunofluorescence. Quantification of EdU positive cells were calculated. **(H,I)** γ-H2AX and 53BP1 staining indicated DSB accumulation. Quantification are represented as means ± SD from at least three independent experiments. A two-sided unpaired *t*-test was used to calculate *P*-values. Significant: *p* < 0.05; NS, not significant: *P* > 0.05.

To confirm the role of RECQ1-PARP1 signaling in mediating GBM cell malignant growth, we transfected a PARP1 overexpression plasmid into RECQ1-depleted M059K cells and evaluated the compensatory influence of PARP1 on cell growth. A protein quantification analysis showed PARP1 downregulation in RECQ1-deficient cells, suggesting that PARP1 function was influenced by decreased RECQ1 abundance, while PARP1 expression increased with PARP1 overexpression plasmid transfection (Figure 5A). A decreased poly(ADP-ribosyl)ation (PARylation) was observed with RECQ1 depletion, while overexpression of PARP restored the modification, which indicated a protective role of PARP1 overexpression with RECQ1 deficiency (Figure 5B). We found that PARP1 overexpression dramatically increased the clonogenic formation capacity of RECQ1-deficient M059K cells after MMS treatment (Figures 5C,D). In agreement with the survival assay, similar results were found through the viability analysis performed by CCK-8 test (Figure 5E). Interestingly, in both EdU labeling and cell cycle assays based on flow cytometry, we consistently found that PARP1 overexpression abolished RECQ1 deficiency-mediated M059K growth inhibition, indicating that the downregulation of PARP1 induced by RECQ1 depletion was a key event in the PARP1 effect on cellular proliferation (Figures 5F,G). Moreover, we also observed dramatically decreased DSB accumulation with PARP1 overexpression, as marked by γ-H2AX and 53BP1 labeling, suggesting that the DNA damage accumulation was largely decreased through PARP1 compensatory activity (Figures 5H,I). Together, our results showed that proficient RECQ1 and PARP1 function in M059K cells was essential for the DNA damage repair induced by MMS. A greater DNA repair capacity can protect M059K cells from overcoming DNA damage that occurred endogenously or upon MMS replication stress, as well as prevent dramatic cellular proliferation.

### RECQ1 Depletion Increased Micronucleus Formation With Hypersensitive to Temozolomide

**FIGURE 6:**
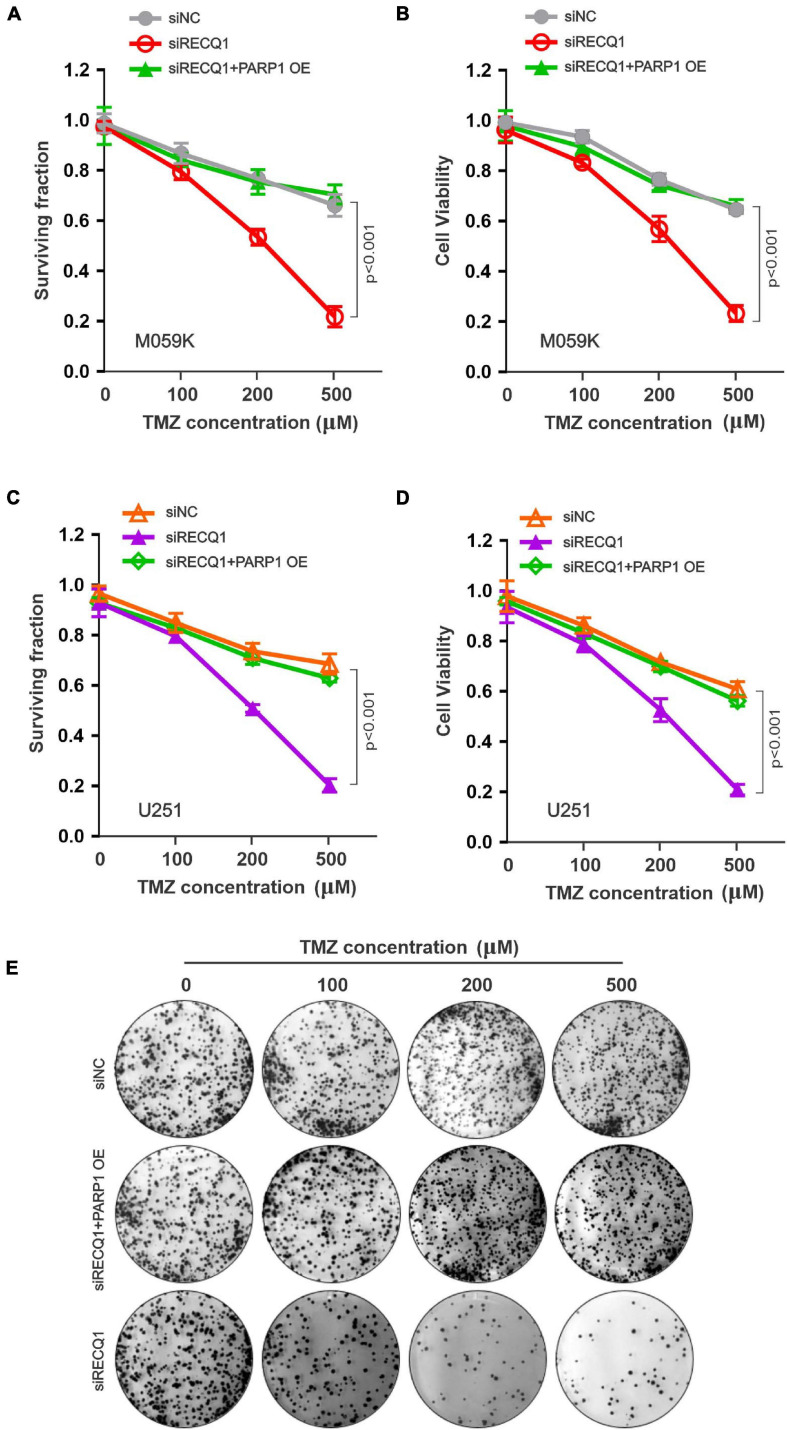
RECQ1 deficient glioblastoma cells are hypersensitive to TMZ treatment. **(A)** Cellular surviving fractions and **(B)** cell viability was measured at different concentration of TMZ in M059K cells transfected with siNC, siRECQ1 or combined with PARP1 OE. Surviving fraction and viability values are indicated as mean ± SD from three independent experiments. **(C,D)** TMZ sensitivity analysis carried out in additional U251 cells. **(E)** Representative clone images were shown. A two-sided unpaired *t*-test was used to calculate *P*-values. Significant: *p* < 0.05; NS, not significant: *P* > 0.05.

Because RECQ1 was found to be overexpressed in glioblastomas, we next investigated the sensitivity of glioblastoma cells to temozolomide (TMZ), which is an anticancer alkylating agent commonly used for the treatment of human brain tumors. Nevertheless, TMZ resistance is a major common challenge to effective clinical therapy (Lefranc et al., 2005, 2007; Stupp et al., 2009). We explored the possibility of RECQ1-PARP1 being a promising signaling target for TMZ hypersensitivity, which would suppress glioblastoma cell proliferation. The surviving cell fraction and viability assays performed with a range of TMZ concentrations showed that RECQ1-depleted M059K cells were hypersensitive to TMZ treatment, while PARP1 overexpression contributed to a robust recovery of cell cycle progression (Figures 6A,B), confirming the role of RECQ1-PARP1 regulation in DNA repair pathways related to DNA replication and chemoresistance. Similar to the effect in M059K cells, knocking down RECQ1 expression sensitized U251 glioblastoma cells to TMZ treatment, while PARP1 supplementation restored GBM cell survival rates and viability (Figures 6C,D), confirming that RECQ1-PARP1 regulation causes significant resistance of glioblastoma cells to the influence of the methylating drug TMZ. Representative clone formation images of U251 cells with the indicated treatment are presented (Figure 6E).

**FIGURE 7:**
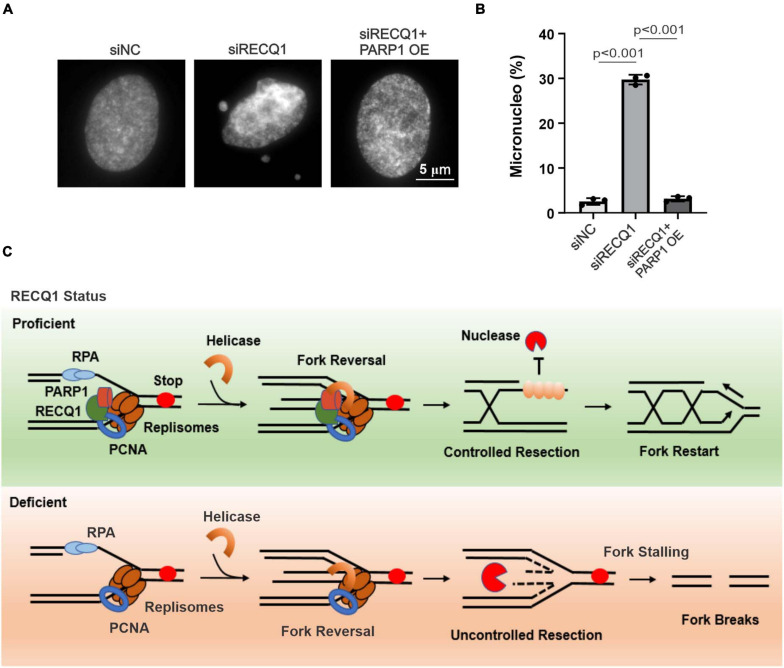
RECQ1 deficient glioblastoma cells raised genomic instability. **(A)** Chromosomal abnormalities were showed by micronucleus formation in M059K cells treated with siRECQ1. **(B)** Quantification are represented as means ± SD from at least three independent experiments. **(C)** Model of RECQ1-PARP1 function. A two-sided unpaired *t*-test was used to calculate *P*-values. Significant: *p* < 0.05; NS, not significant: *P* > 0.05.

Consistent with the micronucleus being a typical sign of genotoxic events and chromosomal instability, RECQ1-deficient cells have been reported to display spontaneous genomic instability (Lucic et al., 2011; Zhang et al., 2015). Thus, we next aimed to explore whether RECQ1 depletion results in an increase in micronuclei changes in response to TMZ. As expected, there was an approximately twenty-fold increase in micronucleus formation in RECQ1-deficient M059K cells treated with TMZ (Figures 7A,B). Collectively, these data indicated the crucial role of RECQ1 in restoring DNA replication progression and protecting GBM cell genomic integrity. Our study also suggested that glioblastoma cells were reliant on this mechanism to escape clinical treatment through drug resistance; therefore, RECQ1-PARP1 signaling may be a suitable target for GBM tumor therapy.

## Discussion

DNA replication stress is considered a major driving force of genome instability and tumorigenesis promotion (Gaillard et al., 2015; Petropoulos et al., 2019). Rapidly proliferating tumor cells also encounter multiple blockades, including altered DNA secondary structures, oncogene activation, and DNA damage drugs, during replication and are usually reliant on overexpressed DNA damage repair factors to overcome these blockades to maintain cancer cell genome integrity (Hurley, 2001; Prado, 2014; Gupta et al., 2018); therefore, these cells are more susceptible to DNA damage response (DDR) inhibition than normal cells, which maintain full DNA repair capacity and provide potential therapeutic alternative strategies for interfering with these repair processes. Accumulating evidence has shown that RECQ1 is involved in several cellular functions, including DNA repair, cell cycle and growth, telomere maintenance, and transcription (Berti et al., 2013; Lu et al., 2013). Earlier studies demonstrated the unique role of RECQ1 in ensuring chromosomal stability and cancer (Sharma et al., 2006). Selectively highly expressed RECQ1 was identified in multiple GBM cells and might be related to the malignancy of tumors and drug resistance to clinical treatment (Bochman, 2014). Therefore, our findings provide a mechanistic rationale for RECQ1-PARP1 regulation of cellular progression, indicating that it may be a promising therapeutic target for the mitigation of glioblastoma progression.

In this study, we reported that the RECQ helicase family member RECQ1 is crucial for restoring stalled replication forks and maintaining GBM cell genomic stability by regulating PARP1 in response to various DNA replication stresses. Associated with PCNA, RECQ1 accumulates at stalled replication forks and recruits PARP1 to limit unprogrammed nucleolytic degradation and assure effective replication fork restart (Figure 7C). RECQ1 depletion increases ssDNA formation and nascent strand degradation under replication stress conditions. PARP1 deletion led to the acquisition of a phenotype similar to that of RECQ1-deficient cells, with increased replication fork stalling, a significant reduction of cellular proliferation and accumulated DNA damage in GBM cells. Consistent with a previous report showing that RECQ1 is important for HeLa cell proliferation and plays a unique role in the maintenance of genome integrity, our results suggest that RECQ1 is essential for DNA replication fork protection and GBM tumor cell malignant proliferation under replication stress conditions. We also found that RECQ1 depletion results in spontaneous γ-H2AX and 53BP1 foci formation in response to MMS exposure, suggesting that RECQ1 plays an essential role in preventing DNA damage accumulating during DNA replication upon replication stress. In addition, the resistance of GBM cells to chemotherapeutic agents is one of the challenges hindering clinical treatment (Roos et al., 2004; Happold et al., 2012). Our study shows that the hypersensitivity of RECQ1-deficient GBM cells to TMZ supports the notion of RECQ1-PARP1 regulation as a contributor to the resistance of glioblastoma cells to the methylating drug temozolomide and represents a promising target pathway for anticancer therapies that inhibit DNA replication and proliferation of GBM cells.

Other human helicases, such as BLM and WRN, are also upregulated in multiple tumors, driving cancer cells to rapidly proliferate, supporting oncogenic activation and potentially playing distinct functions in driving DNA replication stress. Dysregulation of these genes has been associated with cell genetic disorders related to cancer predisposition or promotion (Sidorova et al., 2013; Orlovetskie et al., 2017). Therefore, to determine whether these helicases play functions incompatible to GBM cell growth or have synthetic effects, further study is needed. Cancer cells might utilize these stress-resistant mechanisms during the process of DNA replication to enhance tumorigenesis and chemoresistance; thus, a better understanding of these important mechanisms may benefit GBM clinical therapy. Therefore, combining the newly identified RECQ1-PARP1 signaling function in genome stability and clinical resistance of GBM cells, further studies to discover how these complexities can be resolved to promote fork restarting and progression and the possible synthetic interactions with other human helicases will provide new insights into the mechanism of GBM progression.

## Data Availability Statement

The raw data supporting the conclusions of this article will be made available by the authors, without undue reservation.

## Author Contributions

JZ and CZ had the initial idea, supervised the experiments, and wrote and revised the manuscript. JZ, ML, and HL designed the experiments. KC, MC, BH, LZ, and SX performed the experiments and analyzed the data. YP reviewed and reedited the manuscript. All authors commented on the manuscript.

## Conflict of Interest

The authors declare that the research was conducted in the absence of any commercial or financial relationships that could be construed as a potential conflict of interest.

## Publisher’s Note

All claims expressed in this article are solely those of the authors and do not necessarily represent those of their affiliated organizations, or those of the publisher, the editors and the reviewers. Any product that may be evaluated in this article, or claim that may be made by its manufacturer, is not guaranteed or endorsed by the publisher.

## References

[B1] Andreassen P. R. Ho G. P. D’Andrea A. D. (2006). DNA damage responses and their many interactions with the replication fork. *Carcinogenesis* 27 883–892. 10.1093/carcin/bgi319 16490739

[B2] Arai A. Chano T. Futami K. Furuichi Y. Ikebuchi K. Inui T. (2011). RECQL1 and WRN proteins are potential therapeutic targets in head and neck squamous cell carcinoma. *Cancer Res.* 71 4598–4607. 10.1158/0008-5472.can-11-0320 21571861

[B3] Berti M. Chaudhuri A. R. Thangavel S. Gomathinayagam S. Kenig S. Vujanovic M. (2013). Human RECQ1 promotes restart of replication forks reversed by DNA topoisomerase I inhibition. *Nat. Struct. Mol. Biol.* 20 347–354. 10.1038/nsmb.2501 23396353 PMC3897332

[B4] Bochman M. L. (2014). Roles of DNA helicases in the maintenance of genome integrity. *Mol. Cell Oncol.* 1:e963429. 10.4161/23723548.2014.963429 27308340 PMC4905024

[B5] Bohr V. A. (2008). Rising from the RecQ-age: the role of human RecQ helicases in genome maintenance. *Trends Biochem. Sci.* 33 609–620. 10.1016/j.tibs.2008.09.003 18926708 PMC2606042

[B6] Brosh R. M. Jr. Bohr V. A. (2007). Human premature aging, DNA repair and RecQ helicases. *Nucleic Acids Res.* 35 7527–7544. 10.1093/nar/gkm1008 18006573 PMC2190726

[B7] Debnath S. Sharma S. (2020). RECQ1 helicase in genomic stability and cancer. *Genes* 11:622. 10.3390/genes11060622 32517021 PMC7348745

[B8] Ellis N. A. Groden J. Ye T. Z. Straughen J. Lennon D. J. Ciocci S. (1995). The Bloom’s syndrome gene product is homologous to RecQ helicases. *Cell* 83 655–666. 10.1016/0092-8674(95)90105-1 7585968

[B9] Fugger K. Mistrik M. Neelsen K. J. Yao Q. Zellweger R. Kousholt A. N. (2015). FBH1 catalyzes regression of stalled replication forks. *Cell Rep.* 10 1749–1757. 10.1016/j.celrep.2015.02.028 25772361

[B10] Futami K. Kumagai E. Makino H. Sato A. Takagi M. Shimamoto A. (2008). Anticancer activity of RecQL1 helicase siRNA in mouse xenograft models. *Cancer Sci.* 99 1227–1236. 10.1111/j.1349-7006.2008.00794.x 18422747 PMC11159650

[B11] Gaillard H. Garcia-Muse T. Aguilera A. (2015). Replication stress and cancer. *Nat. Rev. Cancer* 15 276–289. 25907220 10.1038/nrc3916

[B12] Gangopadhyay N. N. Luketich J. D. Opest A. Landreneau R. Schuchert M. J. (2014). PARP inhibitor activates the intrinsic pathway of apoptosis in primary lung cancer cells. *Cancer Invest.* 32 339–348. 10.3109/07357907.2014.919303 24897387

[B13] Gupta R. Somyajit K. Narita T. Maskey E. Stanlie A. Kremer M. (2018). DNA repair network analysis reveals Shieldin as a key regulator of NHEJ and PARP inhibitor sensitivity. *Cell* 173 923–988.e23. 10.1016/j.cell.2018.03.050 PMC8108093 29656893

[B14] Happold C. Roth P. Wick W. Schmidt N. Florea A. M. Silginer M. (2012). Distinct molecular mechanisms of acquired resistance to temozolomide in glioblastoma cells. *J. Neurochem.* 122 444–455. 10.1111/j.1471-4159.2012.07781.x 22564186

[B15] Hickson I. D. (2003). RecQ helicases: caretakers of the genome. *Nat. Rev. Cancer* 3 169–178. 10.1038/nrc1012 12612652

[B16] Hurley L. H. (2001). Secondary DNA structures as molecular targets for cancer therapeutics. *Biochem. Soc. Trans.* 29 692–696. 10.1042/bst0290692 11709056

[B17] Kitao S. Lindor N. M. Shiratori M. Furuichi Y. Shimamoto A. (1999). Rothmund-thomson syndrome responsible gene, RECQL4: genomic structure and products. *Genomics* 61 268–276. 10.1006/geno.1999.5959 10552928

[B18] Lefranc F. Brotchi J. Kiss R. (2005). Possible future issues in the treatment of glioblastomas: special emphasis on cell migration and the resistance of migrating glioblastoma cells to apoptosis. *J. Clin. Oncol.* 23 2411–2422. 10.1200/jco.2005.03.089 15800333

[B19] Lefranc F. Facchini V. Kiss R. (2007). Proautophagic drugs: a novel means to combat apoptosis-resistant cancers, with a special emphasis on glioblastomas. *Oncologist* 12 1395–1403. 10.1634/theoncologist.12-12-1395 18165616

[B20] Lossaint G. Larroque M. Ribeyre C. Bec N. Larroque C. Decaillet C. (2013). FANCD2 binds MCM proteins and controls replisome function upon activation of s phase checkpoint signaling. *Mol. Cell* 51 678–690. 10.1016/j.molcel.2013.07.023 23993743

[B21] Lu X. Parvathaneni S. Hara T. Lal A. Sharma S. (2013). Replication stress induces specific enrichment of RECQ1 at common fragile sites FRA3B and FRA16D. *Mol. Cancer* 12:29. 10.1186/1476-4598-12-29 23601052 PMC3663727

[B22] Lucic B. Zhang Y. King O. Mendoza-Maldonado R. Berti M. Niesen F. H. (2011). A prominent beta-hairpin structure in the winged-helix domain of RECQ1 is required for DNA unwinding and oligomer formation. *Nucleic Acids Res.* 39 1703–1717. 10.1093/nar/gkq1031 21059676 PMC3061051

[B23] Marechal A. Zou L. (2013). DNA damage sensing by the ATM and ATR kinases. *Cold Spring Harb. Perspect. Biol.* 5:a012716. 10.1101/cshperspect.a012716 24003211 PMC3753707

[B24] Mendoza-Maldonado R. Faoro V. Bajpai S. Berti M. Odreman F. Vindigni M. (2011). The human RECQ1 helicase is highly expressed in glioblastoma and plays an important role in tumor cell proliferation. *Mol. Cancer* 10:83. 10.1186/1476-4598-10-83 21752281 PMC3148559

[B25] Opresko P. L. Cheng W. H. Bohr V. A. (2004). Junction of RecQ helicase biochemistry and human disease. *J. Biol. Chem.* 279 18099–18102. 10.1074/jbc.r300034200 15023996

[B26] Orlovetskie N. Serruya R. Abboud-Jarrous G. Jarrous N. (2017). Targeted inhibition of WRN helicase, replication stress and cancer. *Biochim. Biophys. Acta Rev. Cancer* 1867 42–48. 10.1016/j.bbcan.2016.11.004 27902925

[B27] Ouyang K. J. Woo L. L. Ellis N. A. (2008). Homologous recombination and maintenance of genome integrity: cancer and aging through the prism of human RecQ helicases. *Mech. Ageing Dev.* 129 425–440. 10.1016/j.mad.2008.03.003 18430459

[B28] Petropoulos M. Champeris Tsaniras S. Taraviras S. Lygerou Z. (2019). Replication licensing aberrations, replication stress, and genomic instability. *Trends Biochem. Sci.* 44 752–764. 10.1016/j.tibs.2019.03.011 31054805

[B29] Prado F. (2014). Homologous recombination maintenance of genome integrity during DNA damage tolerance. *Mol. Cell. Oncol.* 1:e957039. 10.4161/23723548.2014.957039 27308329 PMC4905194

[B30] Qu A. Wang H. Li J. Wang J. Liu J. Hou Y. (2014). Biological effects of (125)i seeds radiation on A549 lung cancer cells: G2/M arrest and enhanced cell death. *Cancer Invest.* 32 209–217. 10.3109/07357907.2014.905585 24745612

[B31] Roos W. Baumgartner M. Kaina B. (2004). Apoptosis triggered by DNA damage O6-methylguanine in human lymphocytes requires DNA replication and is mediated by p53 and Fas/CD95/Apo-1. *Oncogene* 23 359–367. 10.1038/sj.onc.1207080 14724564

[B32] Sharma S. Doherty K. M. Brosh R. M. Jr. (2006). Mechanisms of RecQ helicases in pathways of DNA metabolism and maintenance of genomic stability. *Biochem. J.* 398 319–337. 10.1042/bj20060450 16925525 PMC1559444

[B33] Sidorova J. M. Kehrli K. Mao F. Monnat R. Jr. (2013). Distinct functions of human RECQ helicases WRN and BLM in replication fork recovery and progression after hydroxyurea-induced stalling. *DNA Repair* 12 128–139. 10.1016/j.dnarep.2012.11.005 23253856 PMC3551992

[B34] Siitonen H. A. Kopra O. Kaariainen H. Haravuori H. Winter R. M. Saamanen A. M. (2003). Molecular defect of RAPADILINO syndrome expands the phenotype spectrum of RECQL diseases. *Hum. Mol. Genet.* 12 2837–2844. 10.1093/hmg/ddg306 12952869

[B35] Stupp R. Hegi M. E. Mason W. P. van den Bent M. J. Taphoorn M. J. Janzer R. C. (2009). Effects of radiotherapy with concomitant and adjuvant temozolomide versus radiotherapy alone on survival in glioblastoma in a randomised phase III study: 5-year analysis of the EORTC-NCIC trial. *Lancet Oncol.* 10 459–466. 10.1016/s1470-2045(09)70025-7 19269895

[B36] Stupp R. Taillibert S. Kanner A. Read W. Steinberg D. Lhermitte B. (2017). Effect of tumor-treating fields plus maintenance Temozolomide vs Maintenance Temozolomide Alone on Survival in Patients With Glioblastoma: a randomized clinical trial. *JAMA* 318 2306–2316. 10.1001/jama.2017.18718 29260225 PMC5820703

[B37] Thakar T. Leung W. Nicolae C. M. Clements K. E. Shen B. Bielinsky A. K. (2020). Ubiquitinated-PCNA protects replication forks from DNA2-mediated degradation by regulating Okazaki fragment maturation and chromatin assembly. *Nat. Commun.* 11:2147. 10.1038/s41467-020-16096-w PMC7195461 32358495

[B38] Toledo L. I. Altmeyer M. Rask M. B. Lukas C. Larsen D. H. Povlsen L. K. (2013). ATR prohibits replication catastrophe by preventing global exhaustion of RPA. *Cell* 155 1088–1103. 10.1016/j.cell.2013.10.043 24267891

[B39] van der Crabben S. N. Hennus M. P. McGregor G. A. Ritter D. I. Nagamani S. C. Wells O. S. (2016). Destabilized SMC5/6 complex leads to chromosome breakage syndrome with severe lung disease. *J. Clin. Invest.* 126 2881–2892. 10.1172/jci82890 27427983 PMC4966312

[B40] Viziteu E. Klein B. Basbous J. Lin Y. L. Hirtz C. Gourzones C. (2017). RECQ1 helicase is involved in replication stress survival and drug resistance in multiple myeloma. *Leukemia* 31 2104–2113. 10.1038/leu.2017.54 28186131 PMC5629372

[B41] Ying S. Chen Z. Medhurst A. L. Neal J. A. Bao Z. Mortusewicz O. (2016). DNA-PKcs and PARP1 bind to unresected stalled DNA replication forks where they recruit XRCC1 to mediate repair. *Cancer Res.* 76 1078–1088. 10.1158/0008-5472.can-15-0608 26603896 PMC4867494

[B42] Yu C. E. Oshima J. Fu Y. H. Wijsman E. M. Hisama F. Alisch R. (1996). Positional cloning of the Werner’s syndrome gene. *Science* 272 258–262. 8602509 10.1126/science.272.5259.258

[B43] Zeman M. K. Cimprich K. A. (2014). Causes and consequences of replication stress. *Nat. Cell Biol.* 16 2–9. 10.1038/ncb2897 24366029 PMC4354890

[B44] Zhang C. Z. Spektor A. Cornils H. Francis J. M. Jackson E. K. Liu S. (2015). Chromothripsis from DNA damage in micronuclei. *Nature* 522 179–184. 10.1038/nature14493 26017310 PMC4742237

