# Supplementary figures and images for: RECQ1 Promotes Stress Resistance and DNA Replication Progression Through PARP1 Signaling Pathway in Glioblastoma
Source: Front Cell Dev Biol. 2021 Jul 26;9:714868. doi: 10.3389/fcell.2021.714868 (PMC8350743; doi:10.3389/fcell.2021.714868)

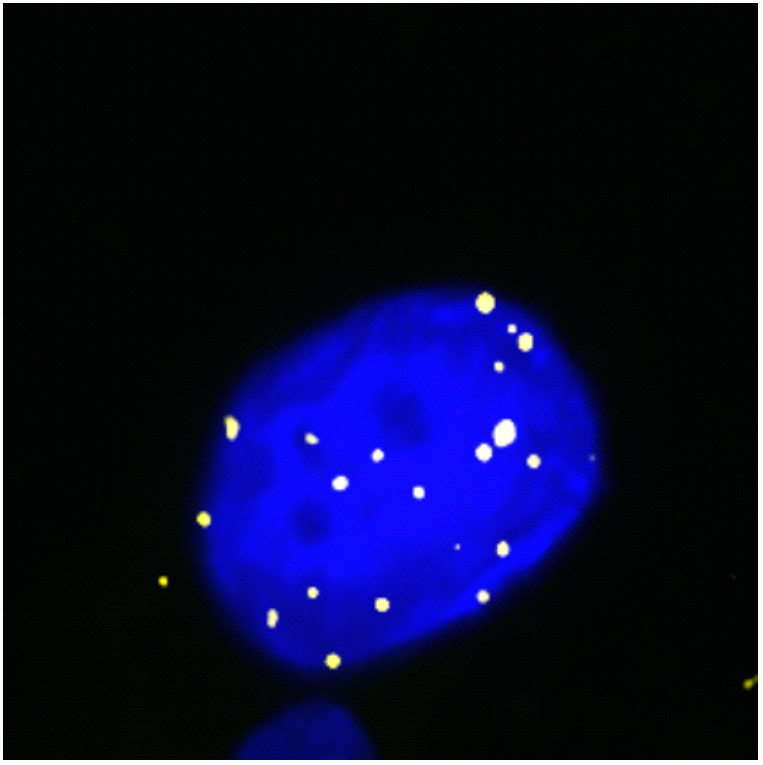

Supplement: Supplementary file 1 [file Data_Sheet_1.ZIP › RAW data/Fig.1B_PLA_MMS.png]

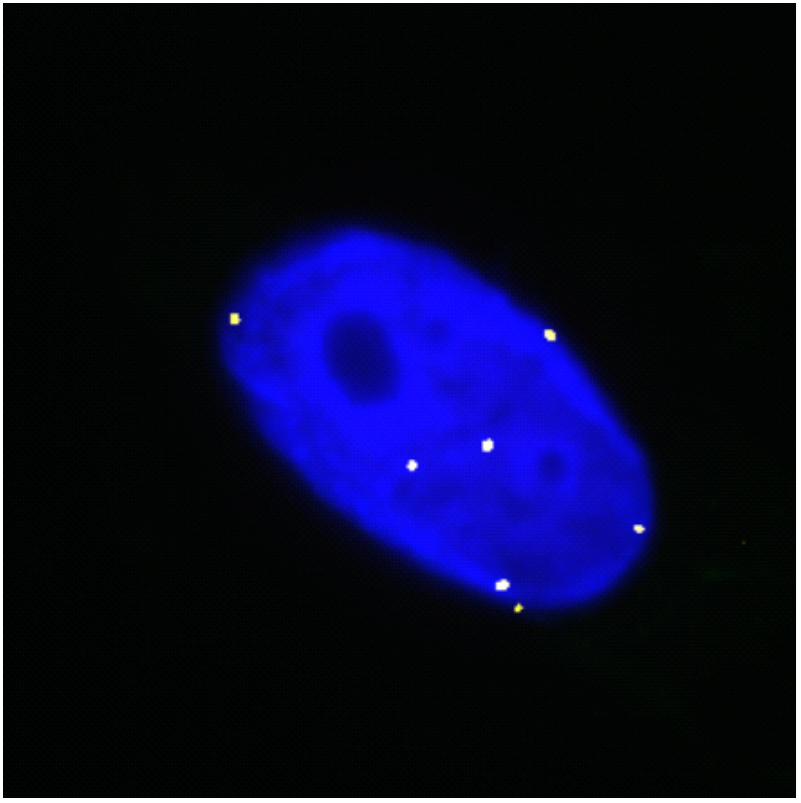

Supplement: Supplementary file 1 [file Data_Sheet_1.ZIP › RAW data/Fig.1B_PLA_NT.png]

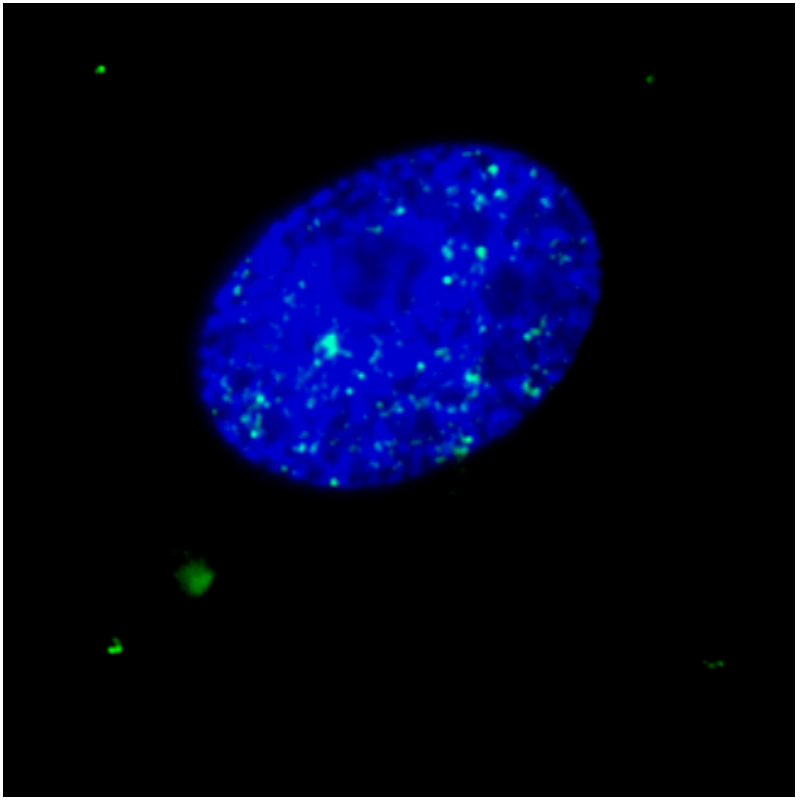

Supplement: Supplementary file 1 [file Data_Sheet_1.ZIP › RAW data/Fig.1C_pRPA_siNC_MMS.png]

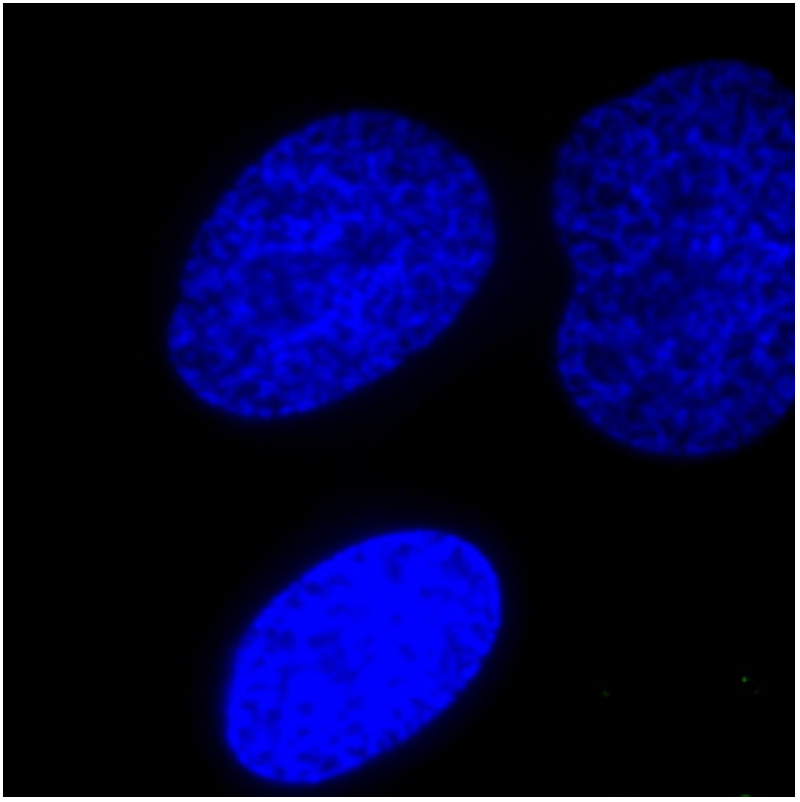

Supplement: Supplementary file 1 [file Data_Sheet_1.ZIP › RAW data/Fig.1C_pRPA_siNC_NT.png]

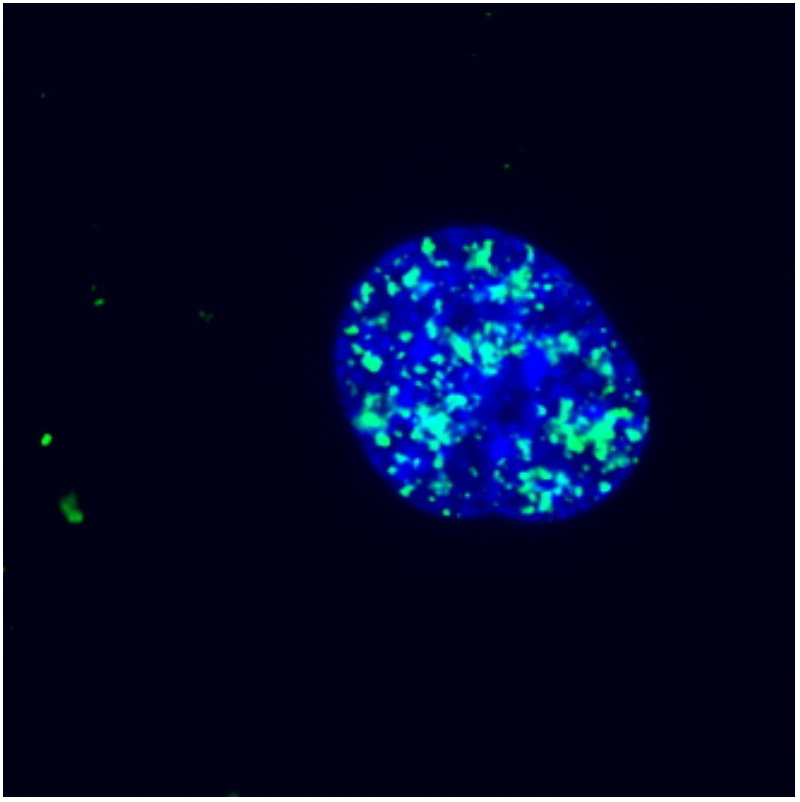

Supplement: Supplementary file 1 [file Data_Sheet_1.ZIP › RAW data/Fig.1C_pRPA_siRECQ1_MMS.png]

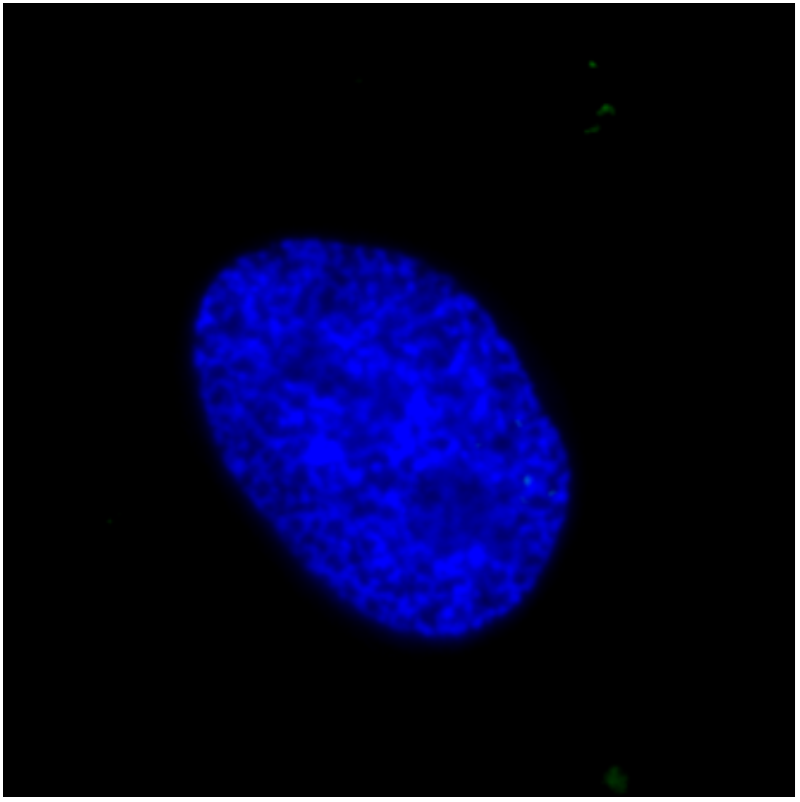

Supplement: Supplementary file 1 [file Data_Sheet_1.ZIP › RAW data/Fig.1C_pRPA_siRECQ1_NT.png]

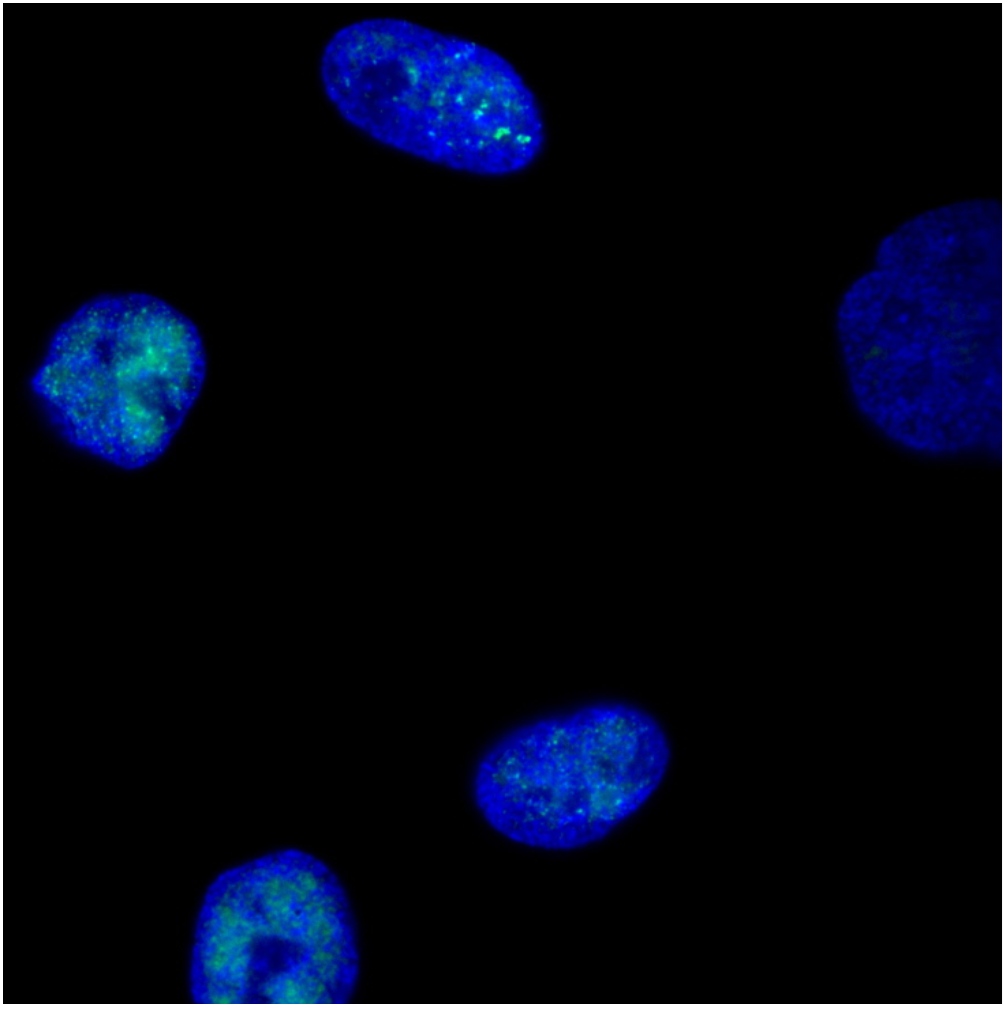

Supplement: Supplementary file 1 [file Data_Sheet_1.ZIP › RAW data/Fig.2F_EdU_siNC_MMS.png]

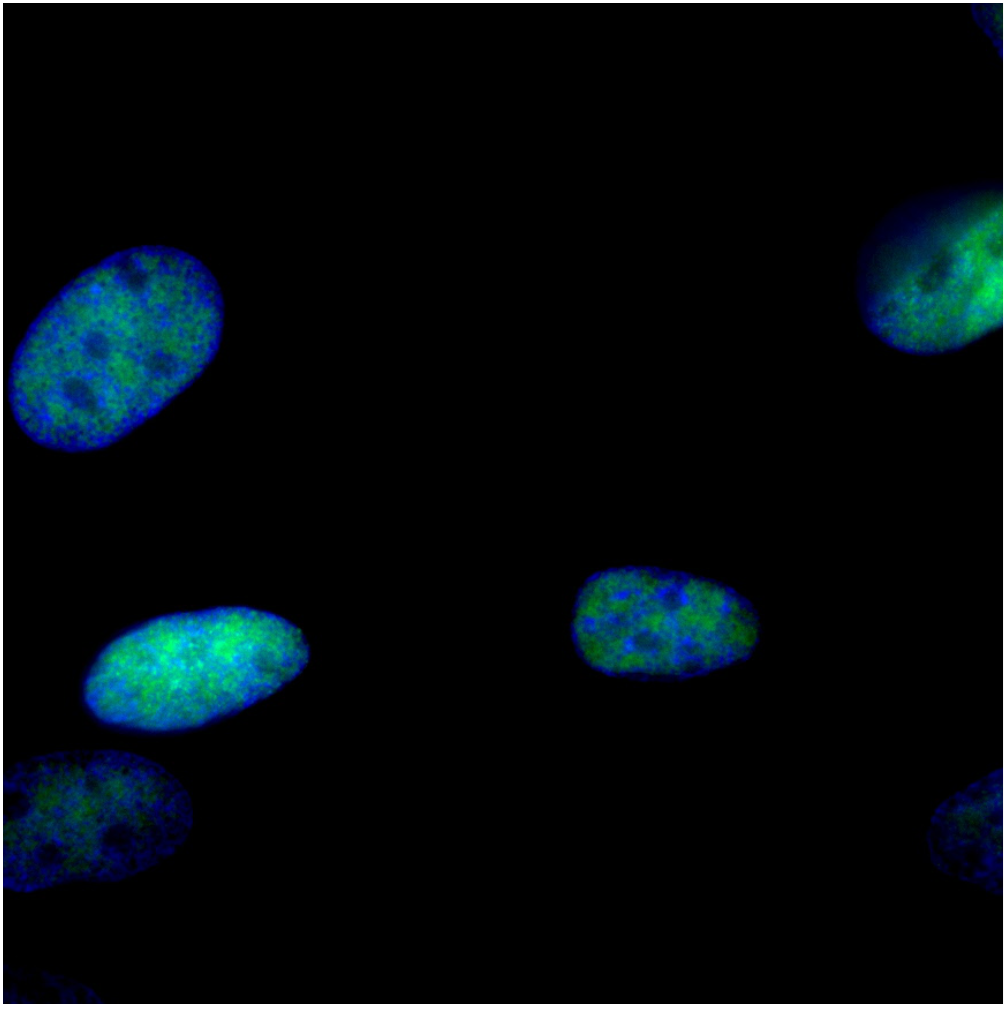

Supplement: Supplementary file 1 [file Data_Sheet_1.ZIP › RAW data/Fig.2F_EdU_siNC_NT.png]

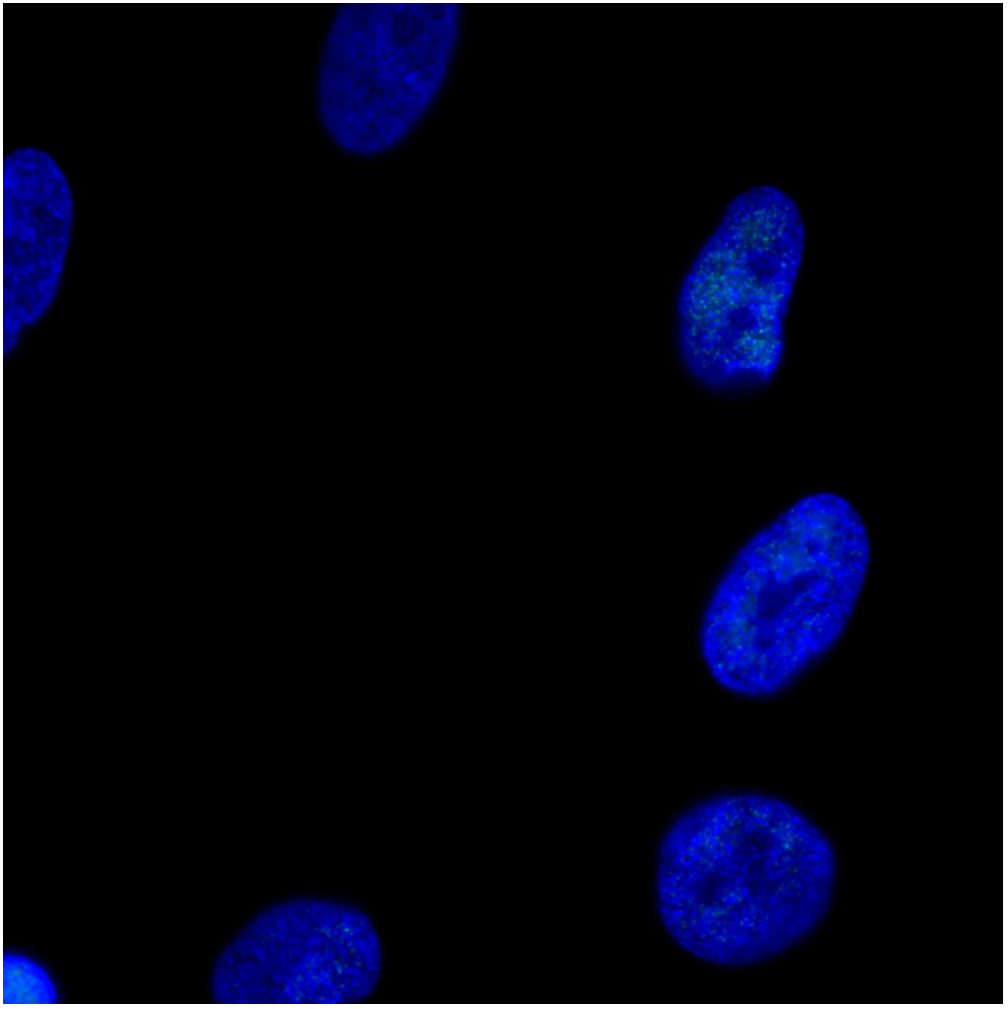

Supplement: Supplementary file 1 [file Data_Sheet_1.ZIP › RAW data/Fig.2F_EdU_siRECQ1_MMS.png]

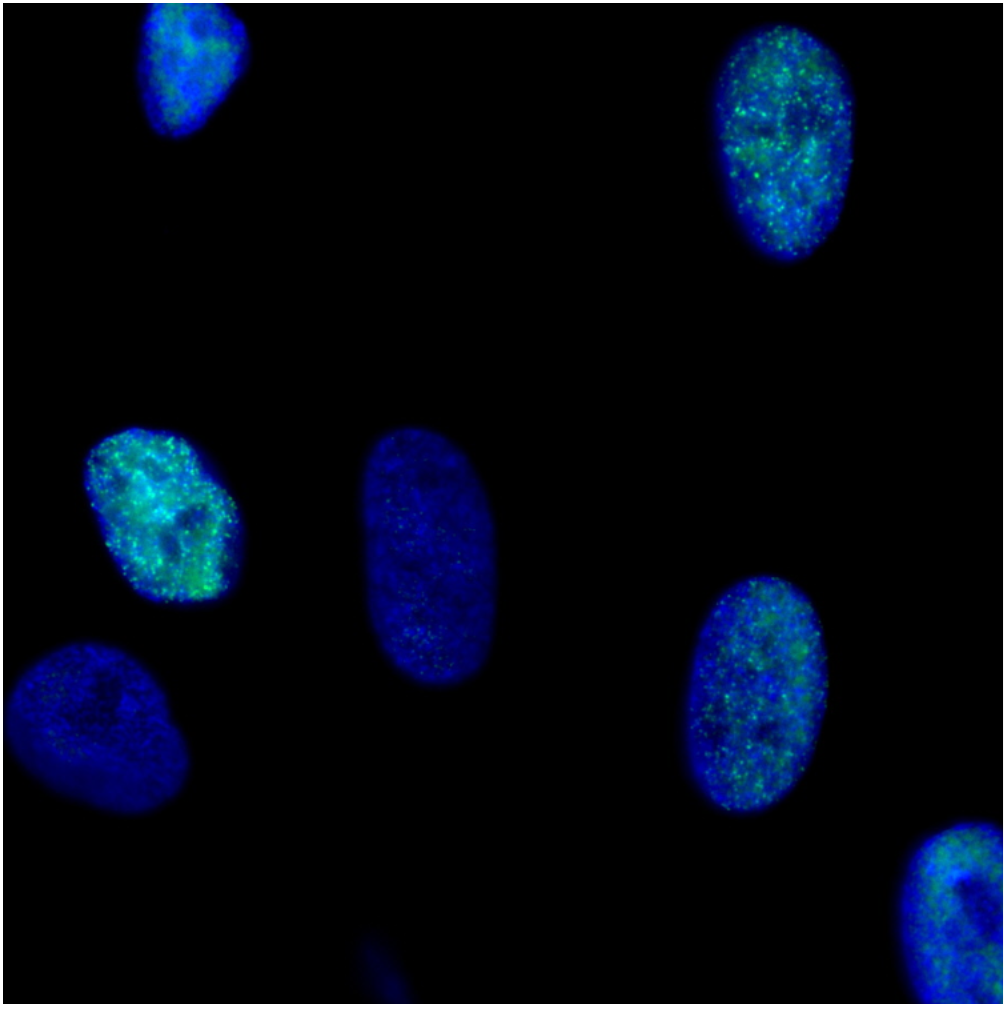

Supplement: Supplementary file 1 [file Data_Sheet_1.ZIP › RAW data/Fig.2F_EdU_siRECQ1_NT.png]

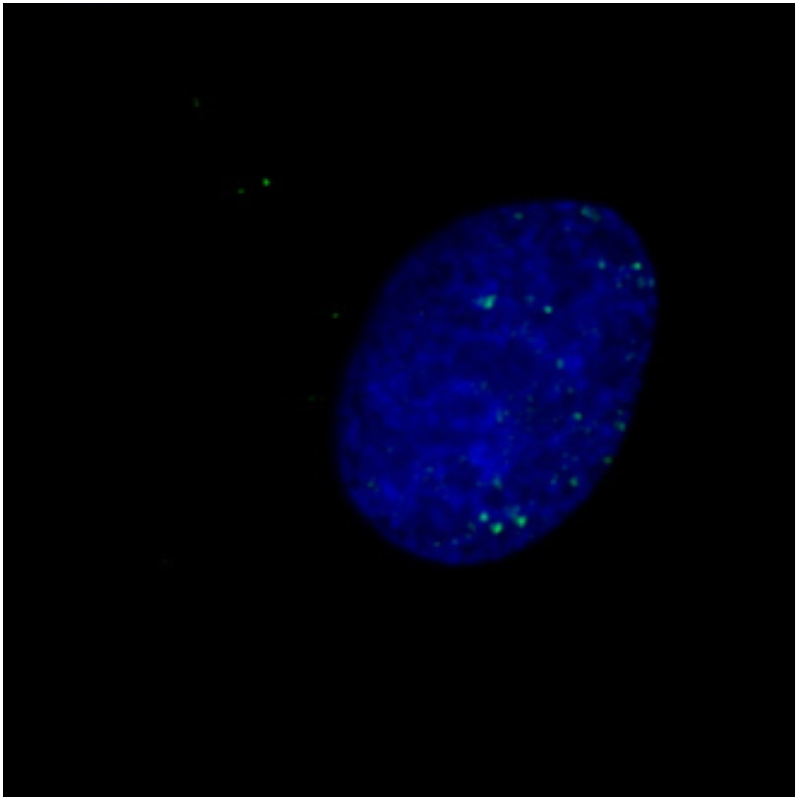

Supplement: Supplementary file 1 [file Data_Sheet_1.ZIP › RAW data/Fig.3A_H2AX_siNC_MMS.png]

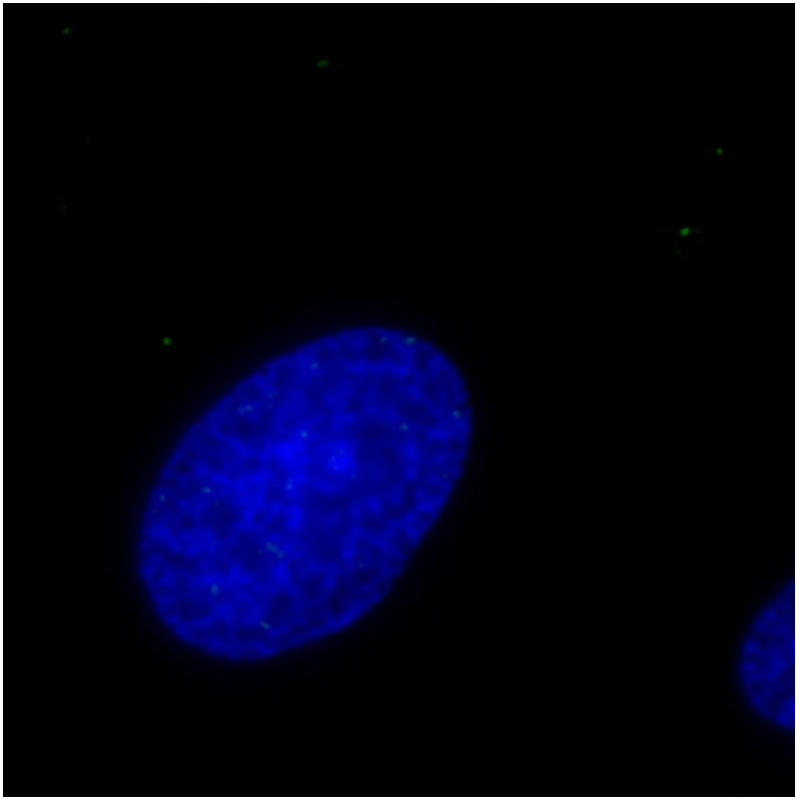

Supplement: Supplementary file 1 [file Data_Sheet_1.ZIP › RAW data/Fig.3A_H2AX_siNC_NT.png]

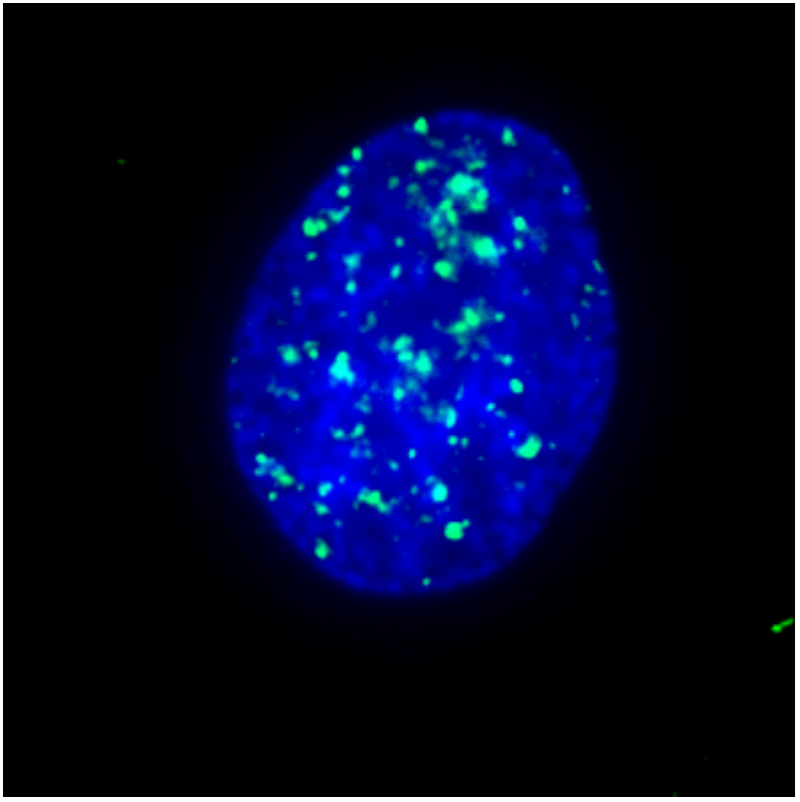

Supplement: Supplementary file 1 [file Data_Sheet_1.ZIP › RAW data/Fig.3A_H2AX_siRECQ1_MMS.png]

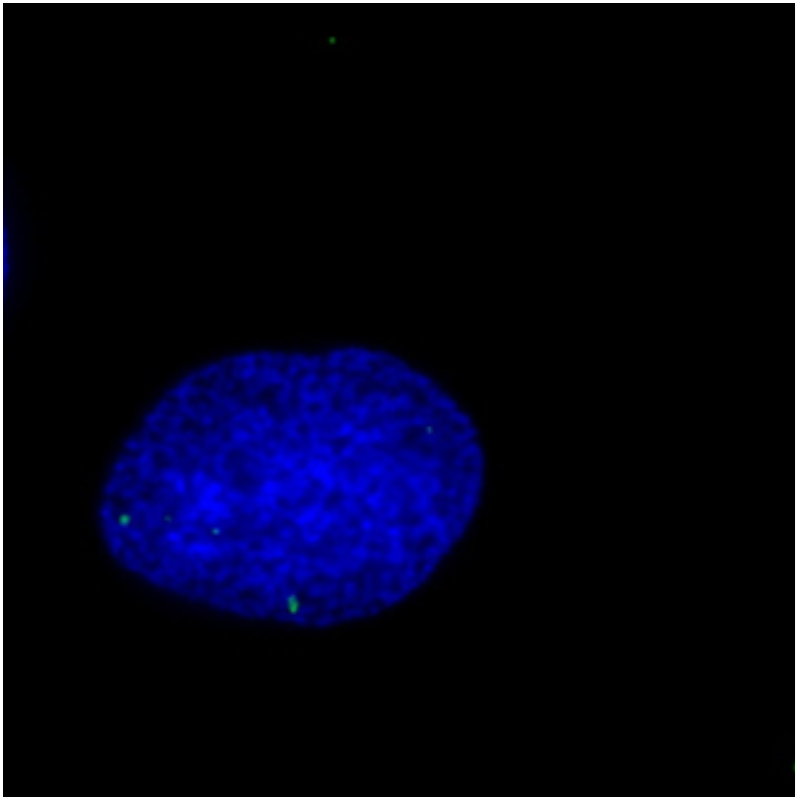

Supplement: Supplementary file 1 [file Data_Sheet_1.ZIP › RAW data/Fig.3A_H2AX_siRECQ1_NT.png]

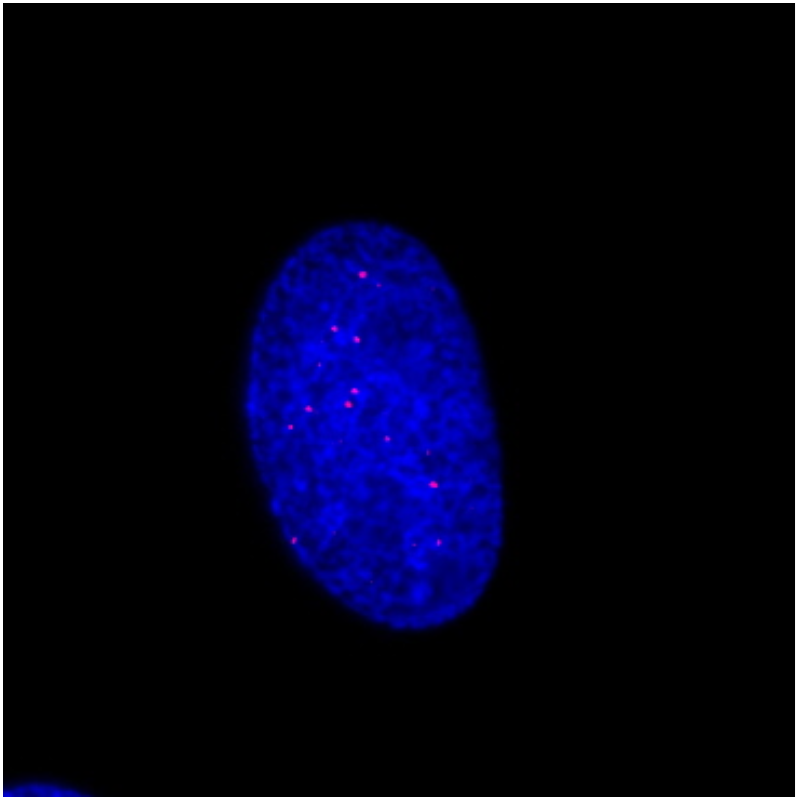

Supplement: Supplementary file 1 [file Data_Sheet_1.ZIP › RAW data/Fig.3B_53BP1_siNC_MMS.png]

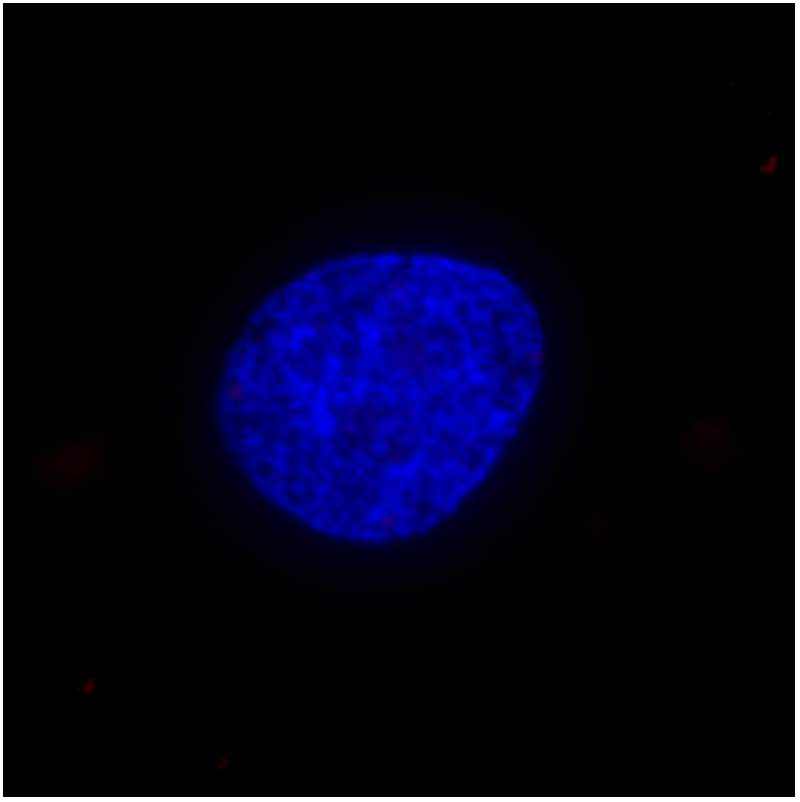

Supplement: Supplementary file 1 [file Data_Sheet_1.ZIP › RAW data/Fig.3B_53BP1_siNC_NT.png]

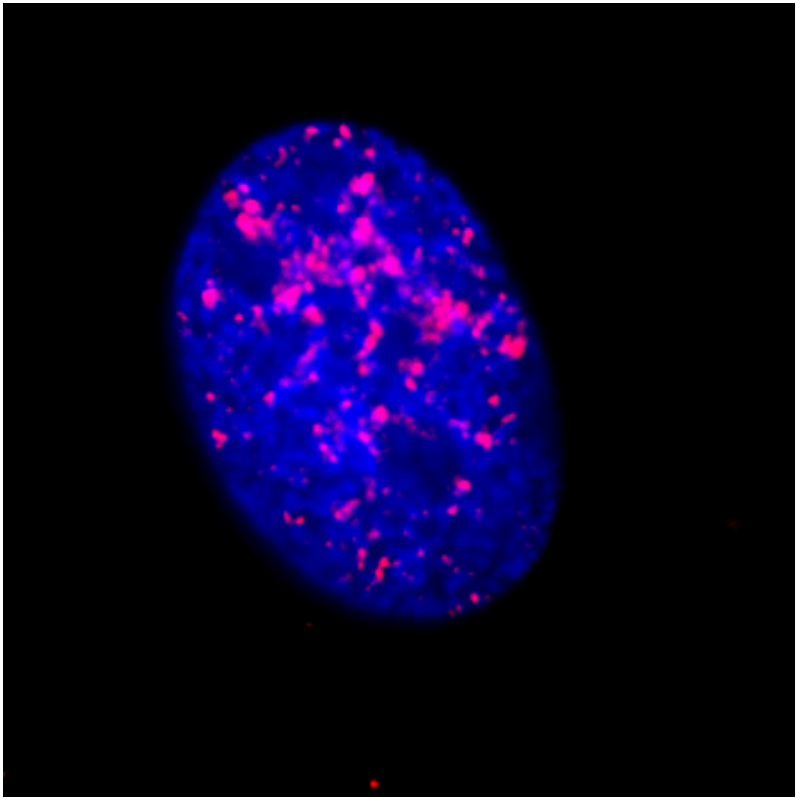

Supplement: Supplementary file 1 [file Data_Sheet_1.ZIP › RAW data/Fig.3B_53BP1_siRECQ1_MMS.png]

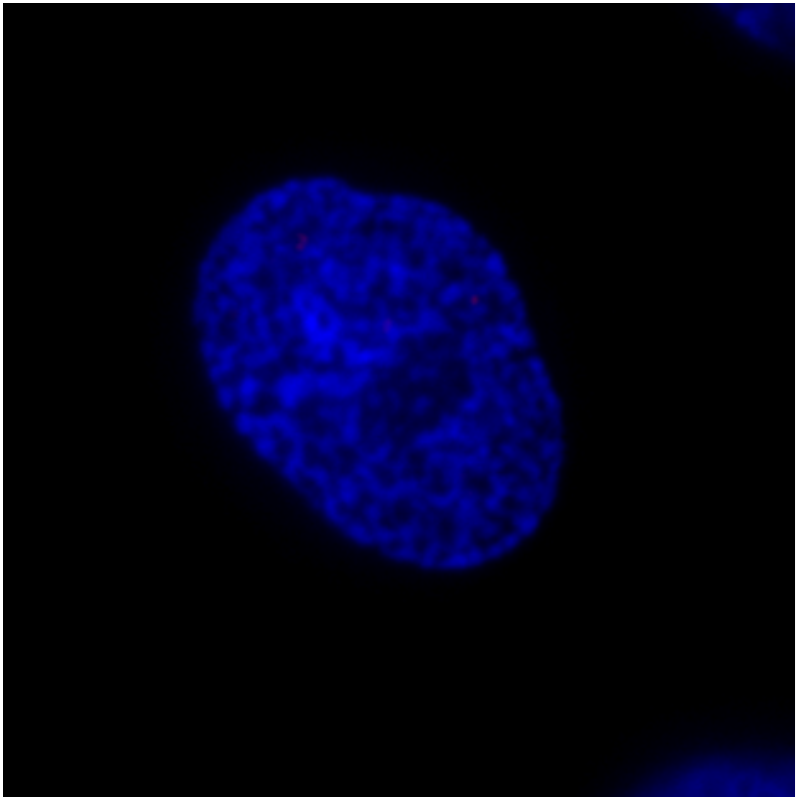

Supplement: Supplementary file 1 [file Data_Sheet_1.ZIP › RAW data/Fig.3B_53BP1_siRECQ1_NT.png]

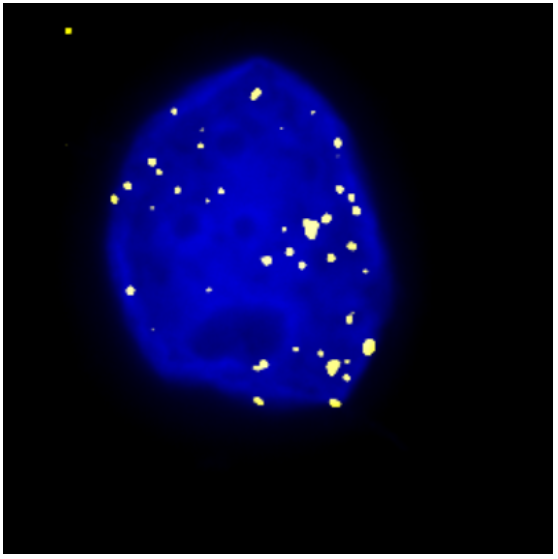

Supplement: Supplementary file 1 [file Data_Sheet_1.ZIP › RAW data/Fig.4A_PLA_siNC_MMS.png]

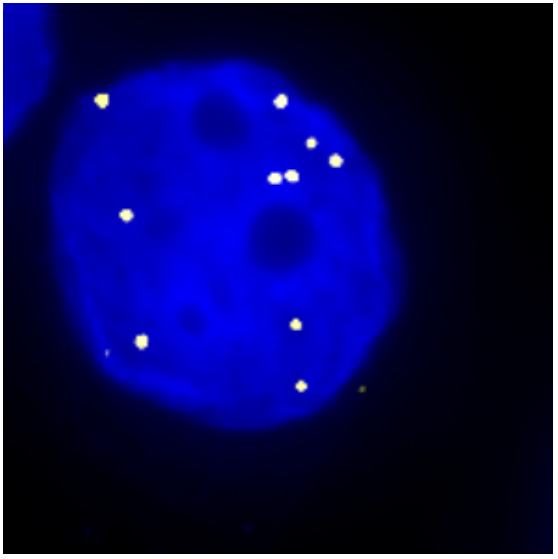

Supplement: Supplementary file 1 [file Data_Sheet_1.ZIP › RAW data/Fig.4A_PLA_siNC_NT.png]

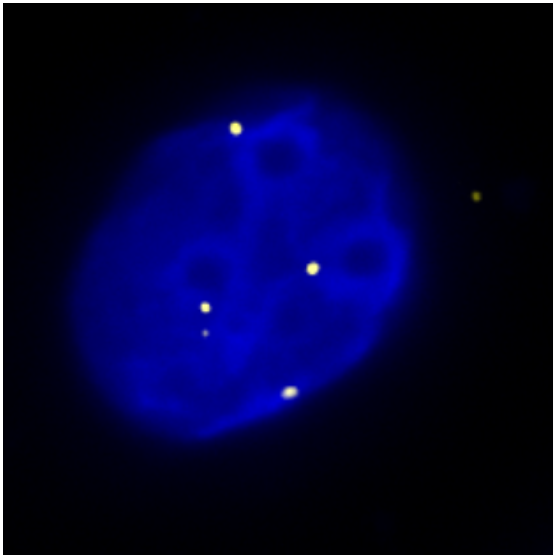

Supplement: Supplementary file 1 [file Data_Sheet_1.ZIP › RAW data/Fig.4A_PLA_siRECQ1_MMS.png]

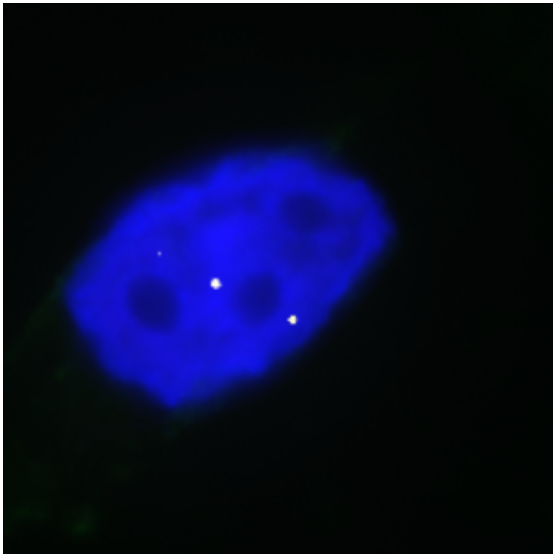

Supplement: Supplementary file 1 [file Data_Sheet_1.ZIP › RAW data/Fig.4A_PLA_siRECQ1_NT.png]

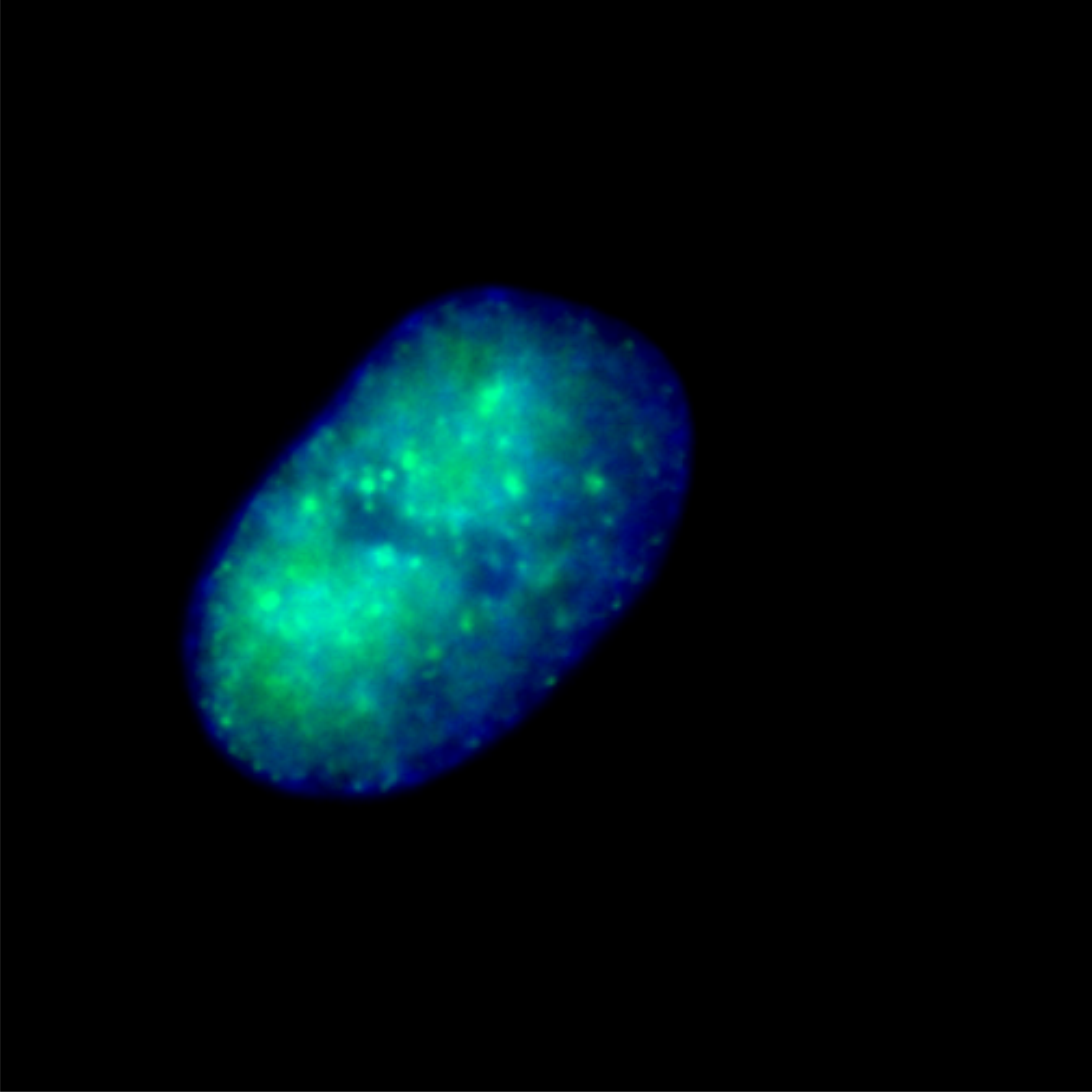

Supplement: Supplementary file 1 [file Data_Sheet_1.ZIP › RAW data/Fig.5B_siNC PAR.png]

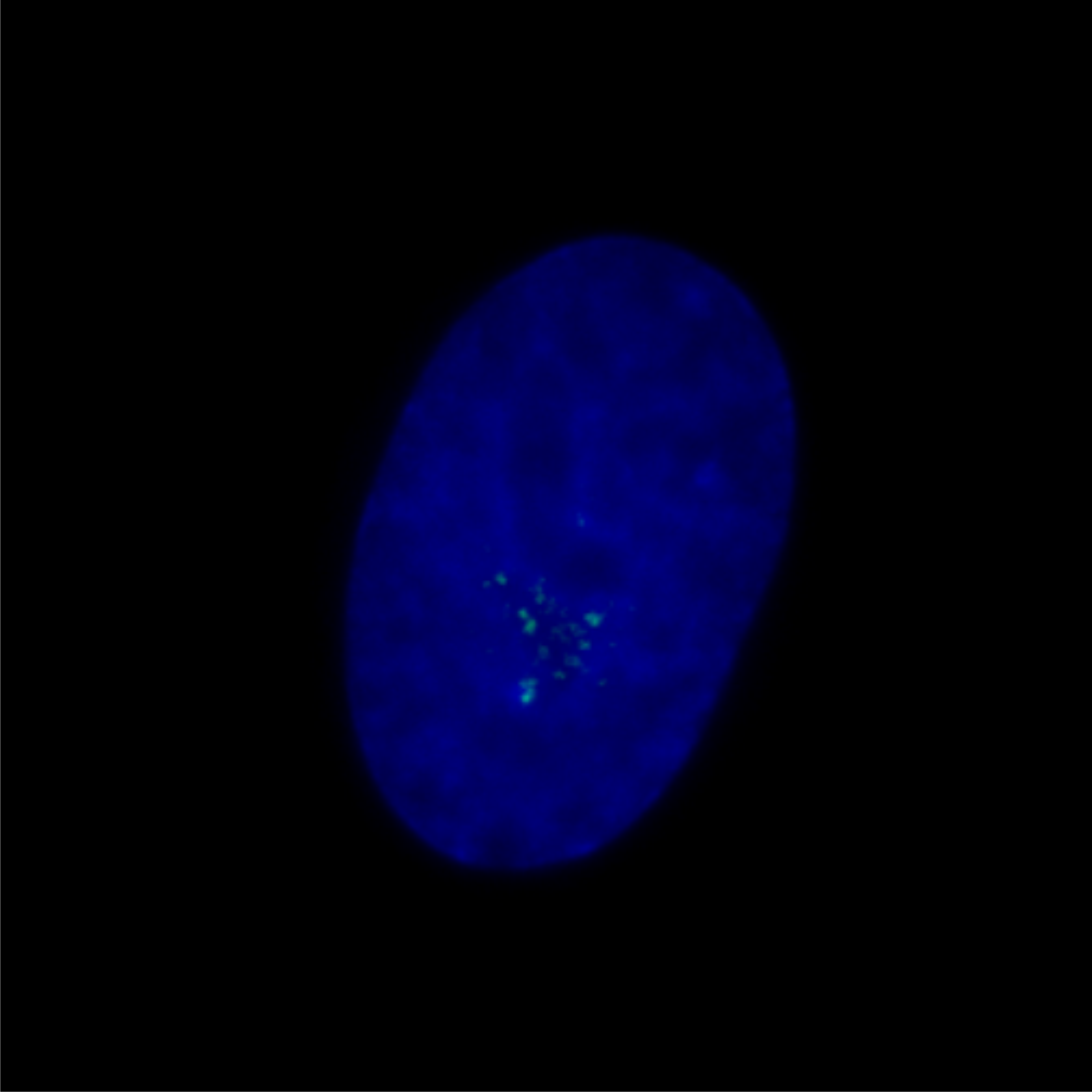

Supplement: Supplementary file 1 [file Data_Sheet_1.ZIP › RAW data/Fig.5B_siRECQ1 PAR.png]

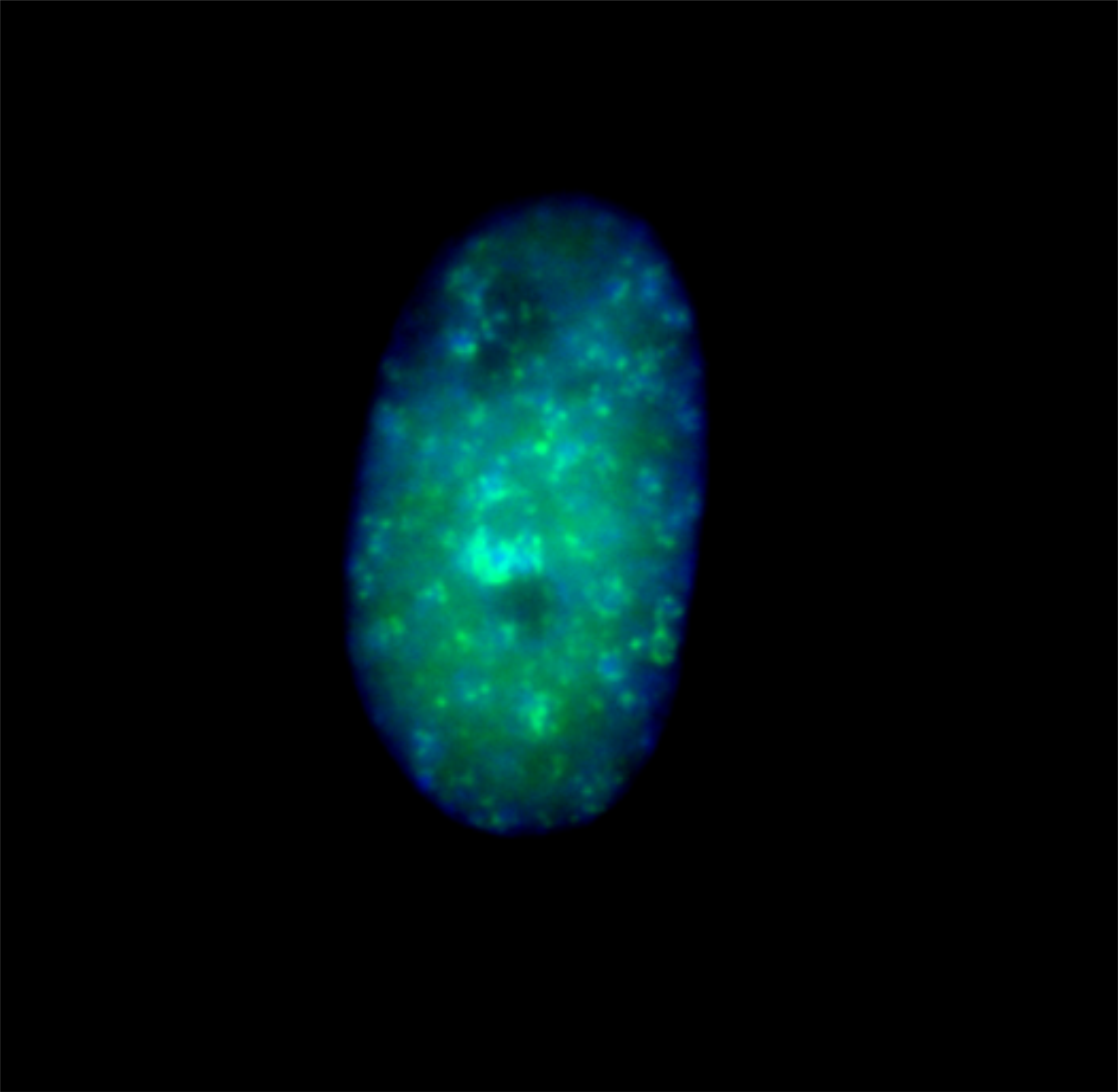

Supplement: Supplementary file 1 [file Data_Sheet_1.ZIP › RAW data/Fig.5B_siRECQ1+PARP OE PAR.png]

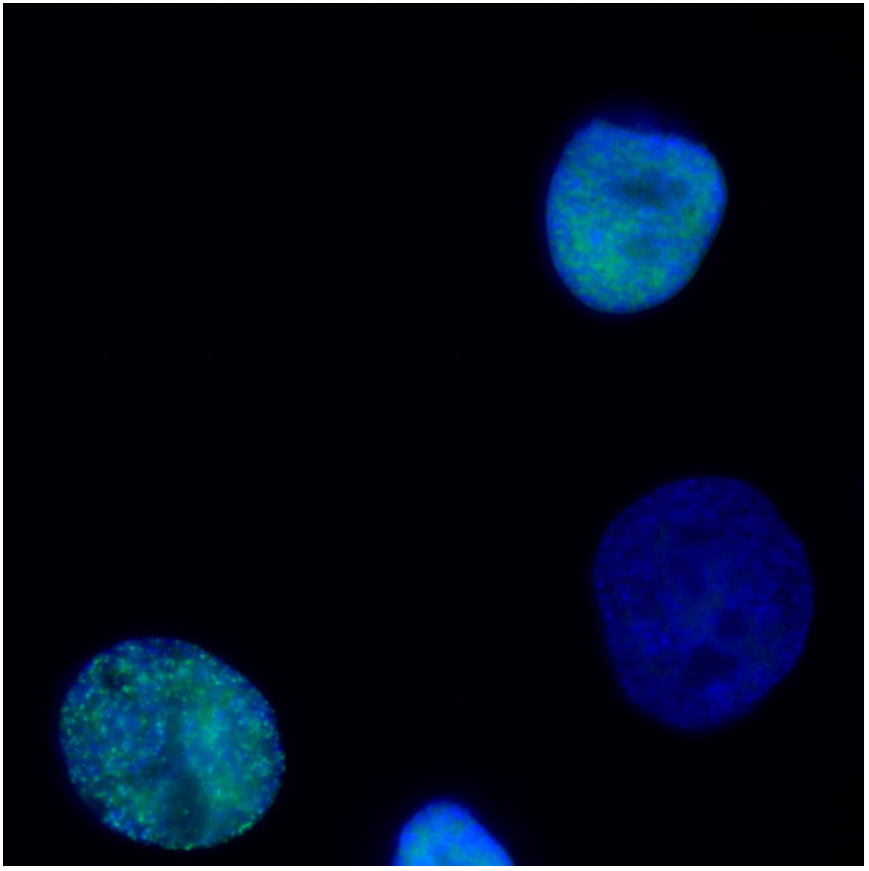

Supplement: Supplementary file 1 [file Data_Sheet_1.ZIP › RAW data/Fig.5F_EdU_siNC.png]

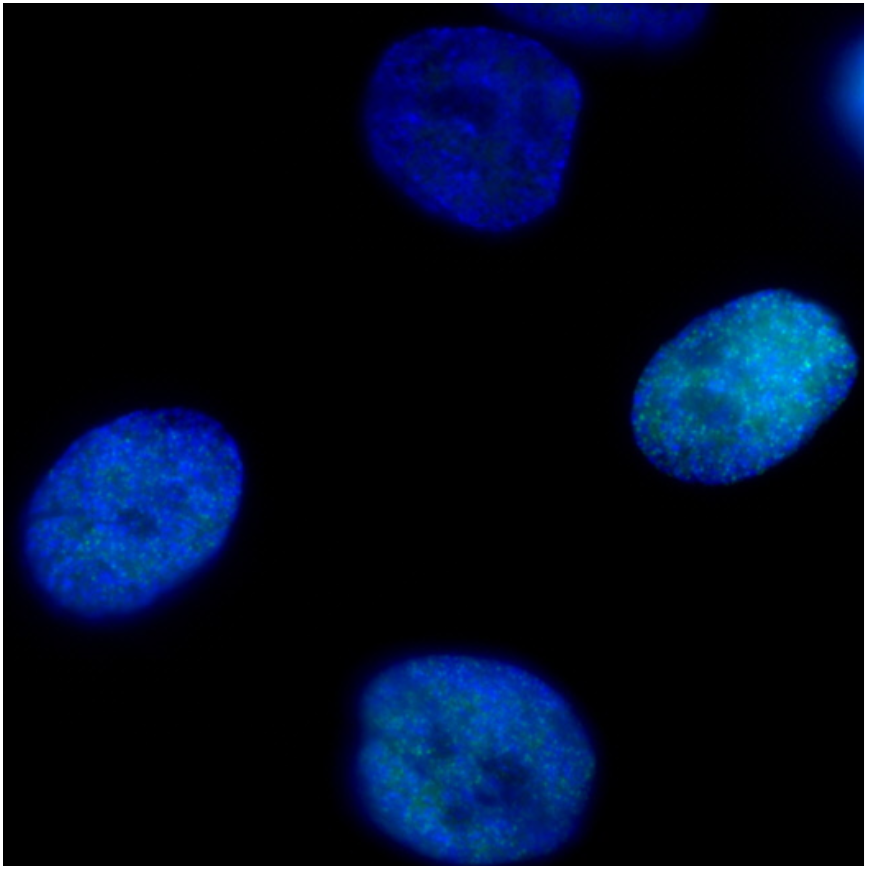

Supplement: Supplementary file 1 [file Data_Sheet_1.ZIP › RAW data/Fig.5F_EdU_siRECQ1+PARP1 OE.png]

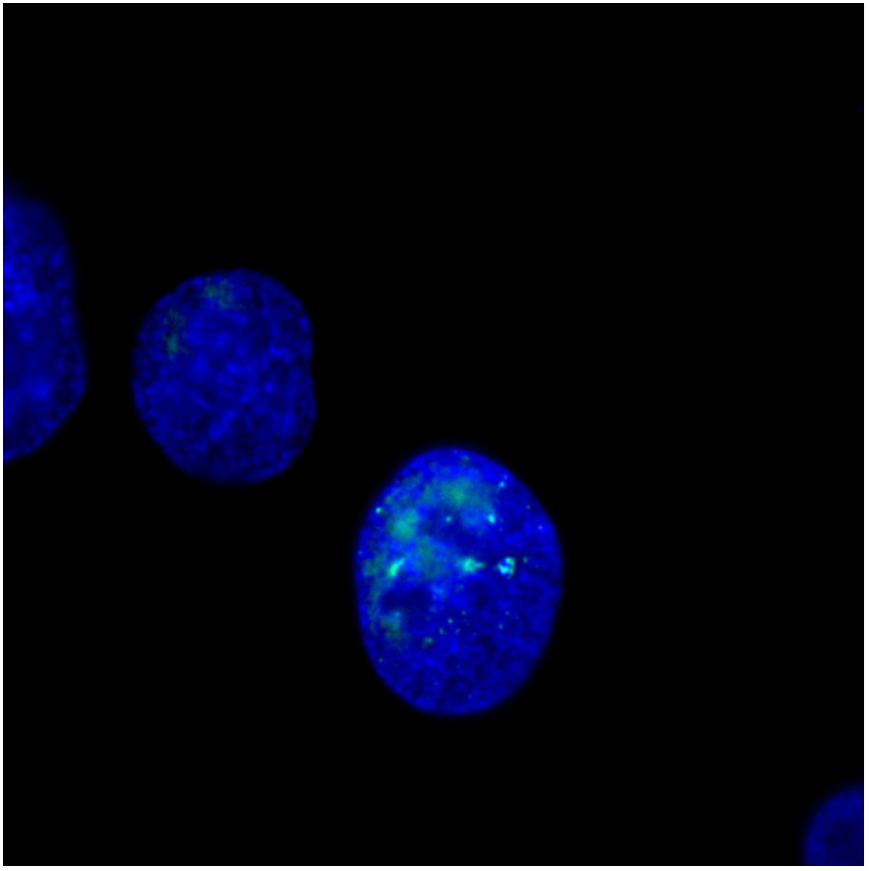

Supplement: Supplementary file 1 [file Data_Sheet_1.ZIP › RAW data/Fig.5F_EdU_siRECQ1.png]

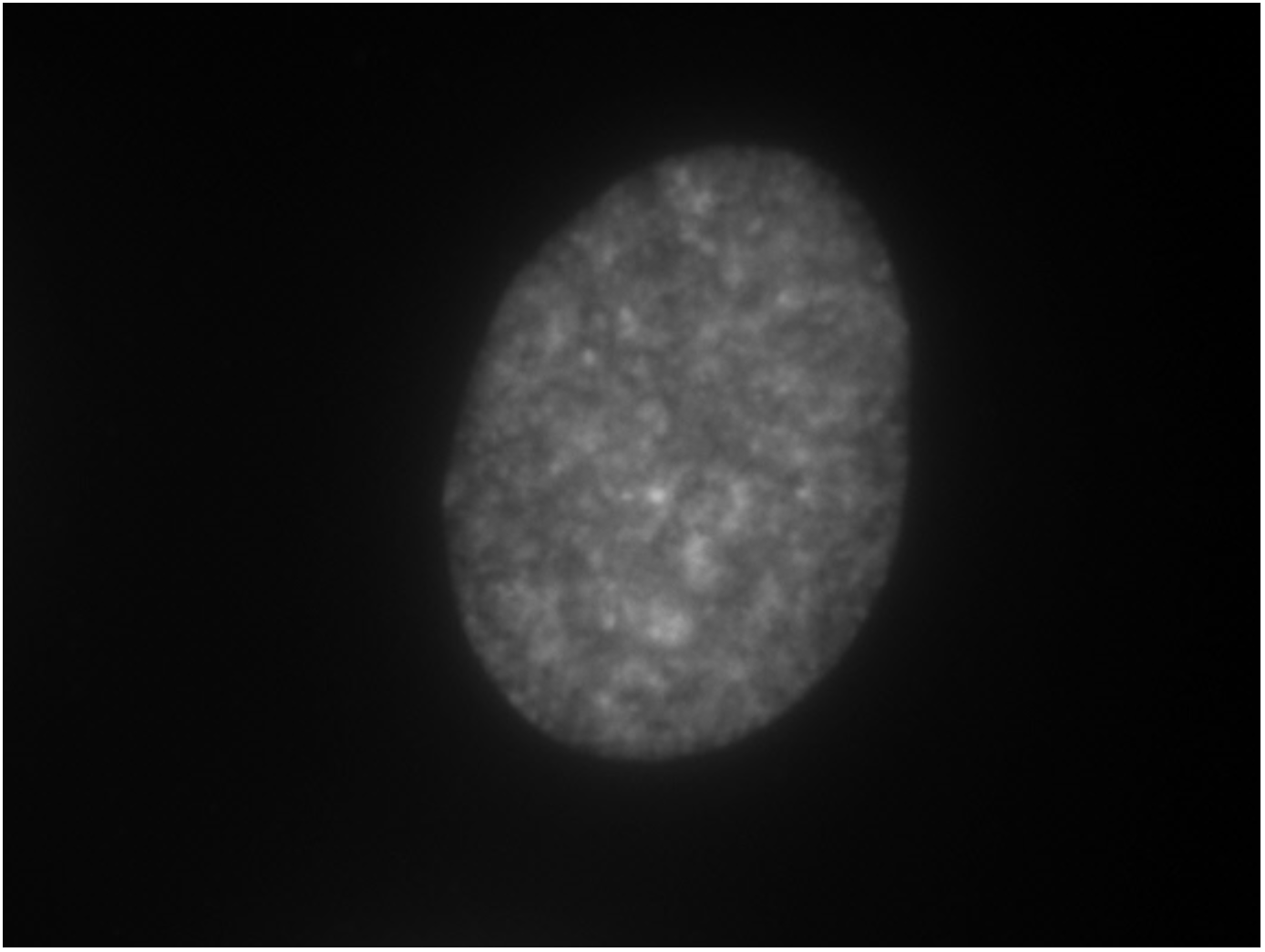

Supplement: Supplementary file 1 [file Data_Sheet_1.ZIP › RAW data/Fig.7 siNC.png]

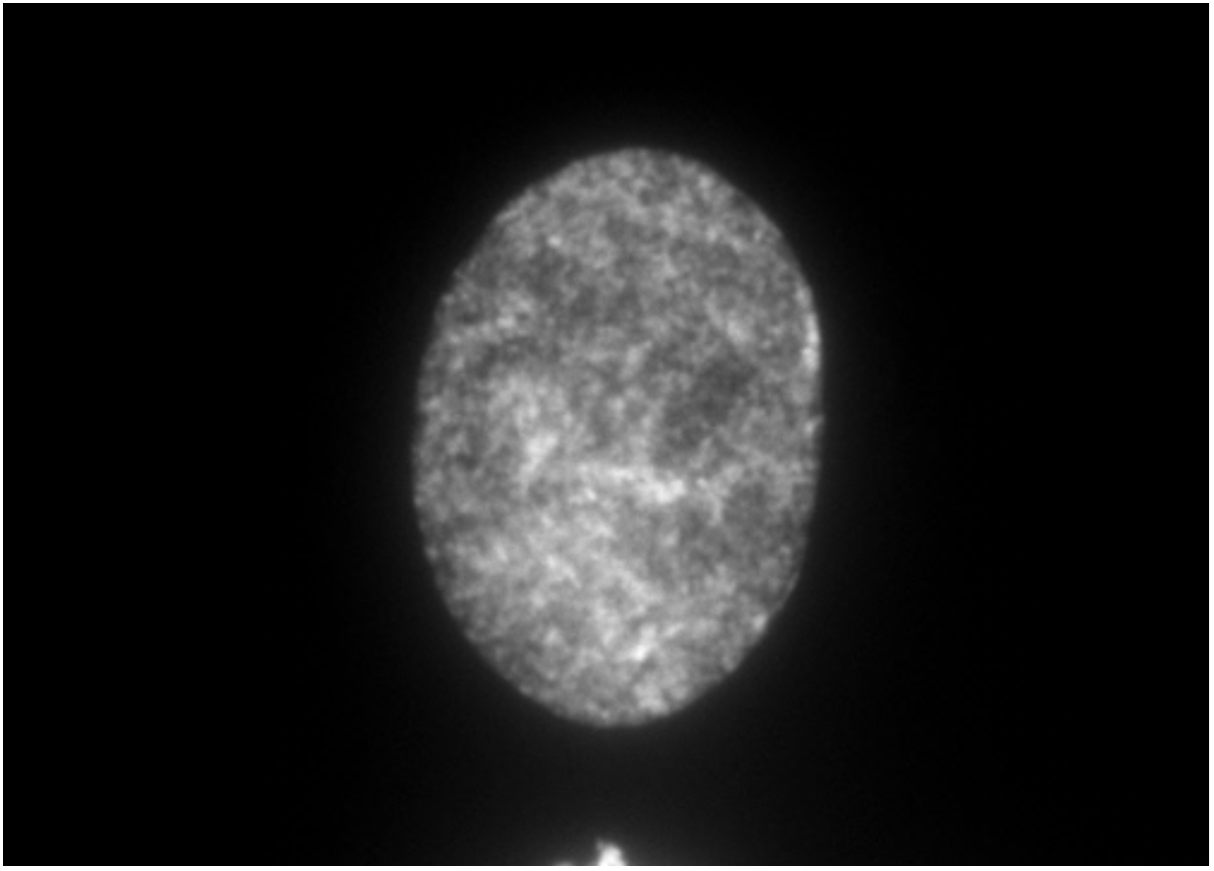

Supplement: Supplementary file 1 [file Data_Sheet_1.ZIP › RAW data/Fig.7_siREC1_PARP1.png]

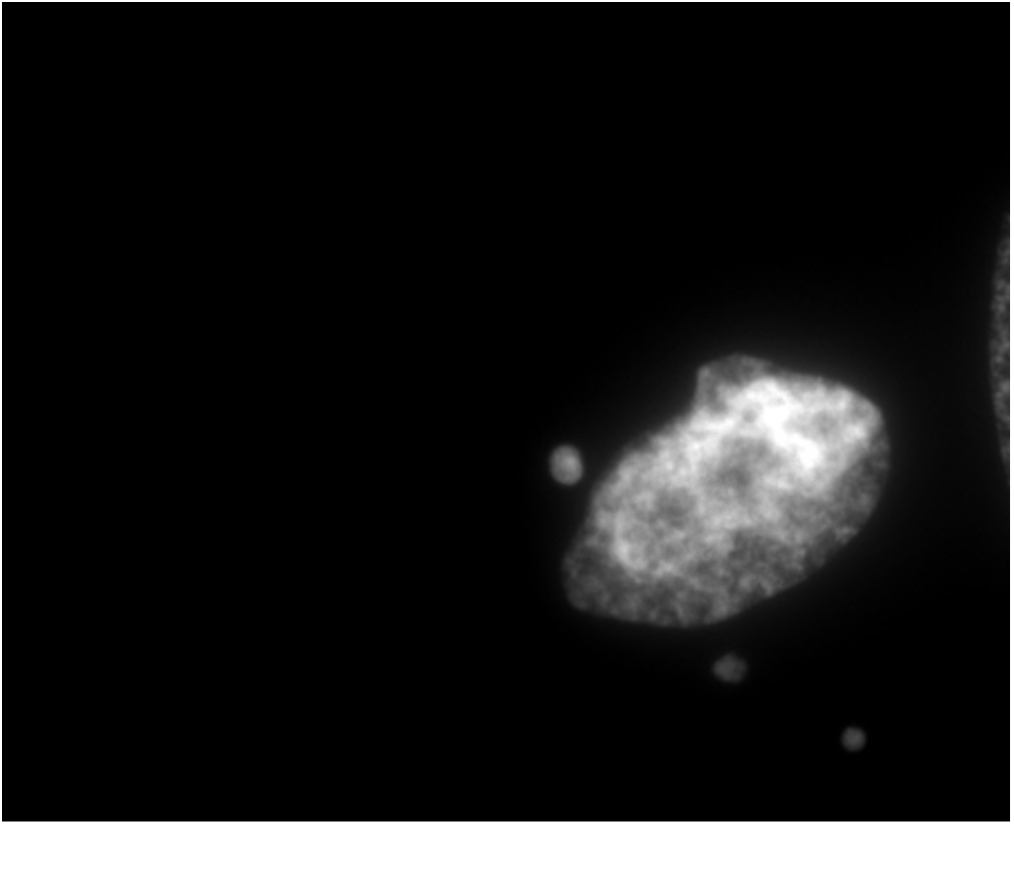

Supplement: Supplementary file 1 [file Data_Sheet_1.ZIP › RAW data/Fig.7_siRECQ1.png]

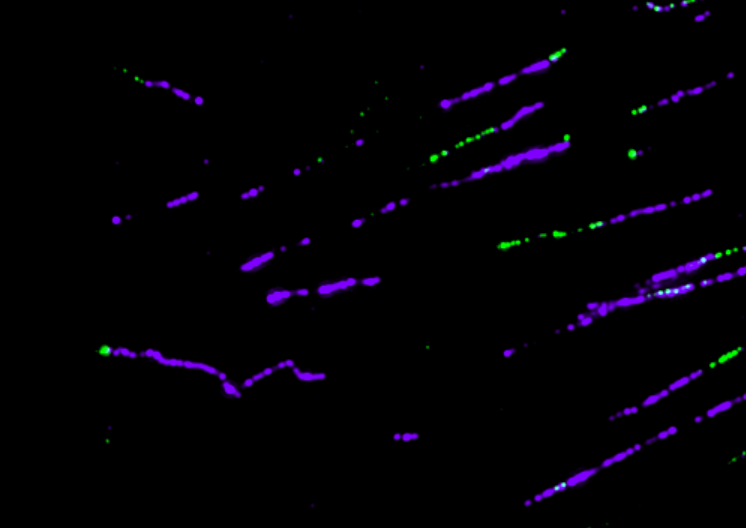

Supplement: Supplementary file 1 [file Data_Sheet_1.ZIP › RAW data/Patterns of fiber_1.png]

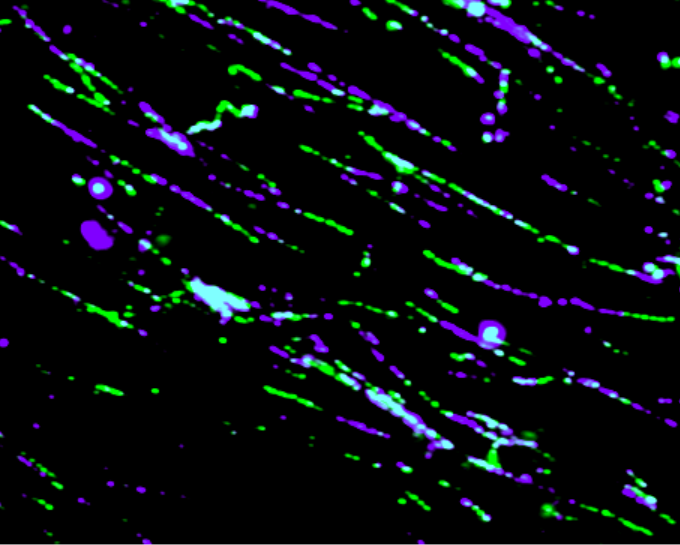

Supplement: Supplementary file 1 [file Data_Sheet_1.ZIP › RAW data/Patterns of fiber_2.png]

Supplementary Figure - Uncropped scans.

Fig.1A

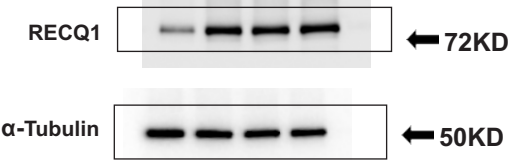

Fig.1D

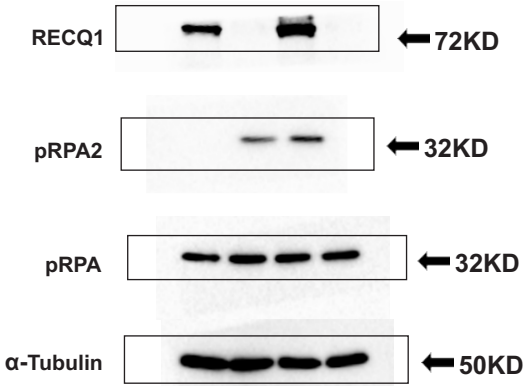

Fig.1E

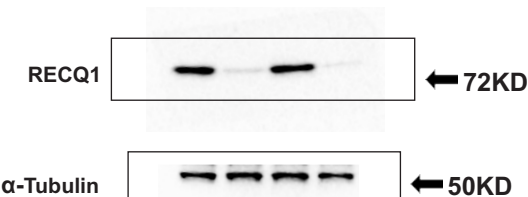

Fig.5A

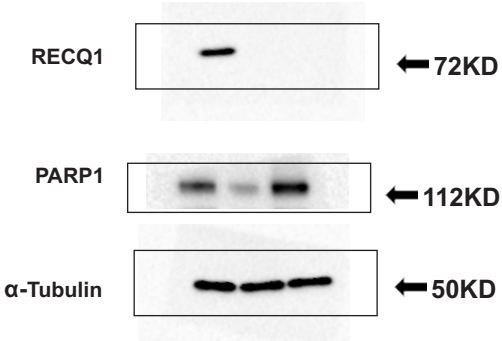

Supplement: Supplementary file 1 [file Data_Sheet_1.ZIP › RAW data/uncropped Western blot scans.pdf]
